# Behavioral Flexibility and the Evolution of Primate Social States

**DOI:** 10.1371/journal.pone.0114099

**Published:** 2014-12-03

**Authors:** Karen B. Strier, Phyllis C. Lee, Anthony R. Ives

**Affiliations:** 1 Department of Anthropology, University of Wisconsin-Madison, Madison, Wisconsin 53705, United States of America; 2 Behaviour and Evolution Research Group, Psychology, School of Natural Sciences, University of Stirling, Stirling, FK9 4LA, United Kingdom; 3 Department of Zoology, University of Wisconsin-Madison, Madison, Wisconsin 53705, United States of America; Midwestern University & Arizona State University, United States of America

## Abstract

Comparative approaches to the evolution of primate social behavior have typically involved two distinct lines of inquiry. One has focused on phylogenetic analyses that treat social traits as static, species-specific characteristics; the other has focused on understanding the behavioral flexibility of particular populations or species in response to local ecological or demographic variables. Here, we combine these approaches by distinguishing between constraining traits such as dispersal regimes (male, female, or bi-sexual), which are relatively invariant, and responding traits such as grouping patterns (stable, fission-fusion, sometimes fission-fusion), which can reflect rapid adjustments to current conditions. Using long-term and cross-sectional data from 29 studies of 22 species of wild primates, we confirm that dispersal regime exhibits a strong phylogenetic signal in our sample. We then show that primate species with high variation in group size and adult sex ratios exhibit variability in grouping pattern (i.e., sometimes fission-fusion) with dispersal regime constraining the grouping response. When assessing demographic variation, we found a strong positive relationship between the variability in group size over time and the number of observation years, which further illustrates the importance of long-term demographic data to interpretations of social behavior. Our approach complements other comparative efforts to understand the role of behavioral flexibility by distinguishing between constraining and responding traits, and incorporating these distinctions into analyses of social states over evolutionary and ecological time.

## Introduction

Evolutionary biologists have spent the last 35 years modeling ecological and demographic influences on sociality. Some of the earliest models of social evolution were developed with respect to non-human primates [1]–[3], taxa which present unique social characteristics in that the majority of the higher primates live in consistent groupings with males and females co-occurring in contrast to strepsirrhines and most other mammals. These distinctive traits have produced an array of ecological models of mating systems, female distributions, and within-group and between-group competitive regimes that have influenced our comparative understanding of social evolution of other organisms, including birds [4], [5], ungulates [6], [7] and social carnivores [8], [9]. Considering their central role in this history, what additional comparative insights might be gained from incorporating the growing number of detailed, long-term field studies on primates into more contemporary models of social evolution?

One of the challenges to addressing this question is distinguishing behavior patterns that are relatively invariant and may constrain other behavioral responses from those that are highly variable and more responsive to local fluctuations in group- and population-wide conditions [10], [11]. Both types of traits may derive from the adaptive history of an organism; however, we distinguish a constraining trait as one that can only change over the very long term or under extreme conditions, while a responding trait is one that is causally related to specific demographic and dispersal predictors, and is facultatively, locally, and temporally variable. This distinction between constraining traits and responding traits is necessary because behavior can both affect and reflect local demographic conditions and the changing social options available to individuals over evolutionary and ecological time [12]–[14].

The relatively slow life histories of primates compared to most other mammals make it likely that during the course of their long lives most individuals will experience an array of social options that reflect dynamic relationships between constraining and responding traits. Long-term studies of wild primate populations provide a unique source of data for evaluating the demographic correlates of individual, intraspecific variation in behavioral traits. These long-term data on demographic and behavioral variation have been examined in particular study groups or populations (e.g., [15], [16]), but among-species comparative analyses of behavioral responses to temporal changes in demographic variation have not yet been applied. Incorporating these data on temporal variation into traditional models of the modal social states [17]–[21], along with recent analyses of behavioral flexibility among extant species [22], has the potential to advance our understanding of social evolution and of the ability of primates to adapt.

We used long-term data compiled from published studies of 29 groups or populations representing 22 species to investigate how two behavioral traits, Dispersal regime (habitually either male-biased, female-biased, or bi-sexual) and Grouping pattern (stable, fission-fusion, and the intermediate condition of sometimes fission-fusion), constrain and respond, respectively, to differences in intraspecific variation in the demographic variables, group size and adult sex ratio. While adult sex ratio can reflect whether there is a single male or multi-male mating structure, these distinctions are more readily explained as reversible phenomena under existing simple models, such as predation risk [23], limits to control of females and male influxes [24], or stochastic alternations [25].

Group size and adult sex ratio have also typically been treated as behavioral traits with modal species values in comparative analyses of primate social and mating systems [11], [25], [26] and social flexibility [22]. However, here we ask how the variation in these demographic variables within one or more groups over time affects grouping patterns under different dispersal regimes. Thus, in our model we disassociated Grouping pattern from demographic variation, and we treated Dispersal regime as a constraining trait because it is well known to be relatively invariant within species in addition to exhibiting a strong phylogenetic signal in primates [11], [18], [27]. We tested the hypotheses that Grouping pattern would respond to demographic variation and that Dispersal regime might constrain these responses.

## Materials and Methods

### Data

We compiled data from 29 studies on 22 species or distinctive subspecies (specifically *Pan troglodytes schweinfurthii* and *P. t. verus*) with published information on group size and composition. The data used in this paper have been previously published in whole or in part, and therefore to our knowledge were collected in compliance with all relevant ethical institutional and governmental permissions. To assemble suitable data sets, we used over 30 years of knowledge of the existing literature on group-forming primate species that have been subjects of long-term field studies; this allowed us to find data that would otherwise have been overlooked in an electronic search. For several well-known studies, sufficient data on demographic variance have not been published, so these studies were necessarily excluded. For several other species, we were either participants in the studies or were able to request data from colleagues. Our data set represents studies of diverse species across the anthropoid primates (with one lemur as an outgroup) Primate Order for which longitudinal or cross-sectional data on group size and sex ratio variance could be accessed.

Four of the studies had detailed data on a single species across 2–21 groups in one population at a single time point (cross-sectional studies). Nine studies had data on 1–29 groups within the same population over 2–20 years, but either provided only mean group sizes or did not follow the same identified groups through time (longitudinal population studies). Finally, 16 studies followed either single groups (8 studies) or 3–11 groups within the same population (8 studies) over 2–31 years (longitudinal group studies). All data are presented in Table S1 and Table S2; references to the data are provided in Table S3.

We assigned a habitual dispersal pattern to each species: Male-biased (n = 10), Female-biased (n = 6), or Bi-sexual (n = 6). These assignments assumed that dispersal is a trait with low variance except during exceptional demographic conditions [28]. Atypical dispersal is common when group densities are low or movement opportunities are restricted, and although variance over time in which sex disperses is an important trait to explore [29], we know of relatively few long-term studies (e.g., Thomas' langurs, [30]; northern muriqui, [31]) or genetic estimates (e.g., chimpanzees [32]; Guereza colobus [33]; red colobus [34]; woolly monkeys and spider monkeys [35]) that could provide data on the proportion of each sex dispersing when typical dispersal patterns are violated.

We classified species' Grouping patterns as: Stable (S, n = 11), when the same individuals aggregate and coordinate activities and movements; Fission-fusion (FF, n = 4), when parties have variable composition and duration; and the intermediate condition (SFF, n = 7) in which a species might exhibit both S and FF at different times or in different populations. SFF groups were typically stable, but smaller units were seen apart from the main group; such groups would show generally high levels of consistency in their associations, but intermediate levels of temporal aggregation [36]. We considered S and FF to be invariant, and SFF to be the variant condition most likely to respond to demographic variation, at least within the constraints imposed by Dispersal.

Group size is a difficult variable for observers to determine and report, in part because births, deaths, immigrations, and emigrations (whether seasonal or randomly distributed) affect group size in both the short and the longer term. We have assumed that group size is an inherently dynamic trait. To measure variation in group size, we used the coefficient of variation (CV = standard deviation/mean), which scales the variation according to the mean. For the 4 cross-sectional studies, the CV in group size measures variation among groups at a single time point. For the 9 longitudinal population studies, the CV in group size is the variation in mean group sizes through time (if only mean group sizes are given) or the variation in size of all study groups. For the 16 longitudinal group studies, the CV was calculated through time for each group in the study population, and then the average CV was calculated among groups when more than one group was described. We measured variation in the adult sex ratio similarly to variation in group size, although rather than the CV, we used the variance in arcsine-square root transformed proportion of males in the population.

We obtained a phylogeny for the study species from the 10k Trees Website [37]. All study species were available except *Brachyteles hypoxanthus* for which we substituted *Brachyteles arachnoides* without changing the tree.

### Analyses

We used Ancestral Character Estimation [38]–[40] to compute the maximum likelihood of the distribution of the Dispersal trait across the phylogeny. To statistically assess phylogenetic signal, we used a likelihood ratio test to compare this maximum likelihood to the maximum likelihood estimated assuming all species are independent (i.e., a “star” phylogeny); because the phylogenetic signal is bounded by zero, the likelihood ratio is distributed by a χ^2^_0_+χ^2^_1_ distribution [41]. We used models of trait evolution that either assumed the same transition rates among states or state-specific transition rates; both models gave the same statistical results, so we only present the results of the simpler model assuming the same transition rates for all traits.

To determine if Dispersal constrains Grouping, we tested whether Dispersal predicts the Grouping status FF versus S+SFF, and then the Grouping status S versus FF+SFF. In other words, the analyses test whether Dispersal traits predict species that never have FF or never have S. We use this approach to account for SFF being the intermediate state between S and FF. These analyses were performed using phylogenetic logistic regression [42], which simultaneously computes the phylogenetic signal in the residuals after accounting for the effect of the predictor variable, Dispersal. Because we treat Grouping as a binary trait we cannot compute phylogenetic rates of change in these models [42], [43].

For studies with longitudinal data, we might expect effects of either the duration of the study or the number of observations on the CV in group size. To evaluate this possibility, we regressed CV in group size for the longitudinal data sets against log_10_ number of observations and used the residuals from this regression; for studies with multiple groups, we used the greatest number of observations for a single group. For the 7 species that were subjects of 2 studies, the residual values of the CVs were calculated for each study separately and then averaged. To control for differences in the CVs in group size among the three types of data sets (longitudinal on groups, longitudinal on populations with group sizes averaged, and cross-sectional), the CVs within each type of data were separately standardized to have mean zero and variance one. We performed a similar analysis of the effect of study duration on the variation in sex ratio. Regressions of Grouping on CV in group size and variation in sex ratio were performed with phylogenetic logistic regression [42]. The correlation between CV in group size and variation in sex ratio was low (Pearson correlation coefficient = 0.35), so we performed these analyses separately.

## Results

**Figure 1:**
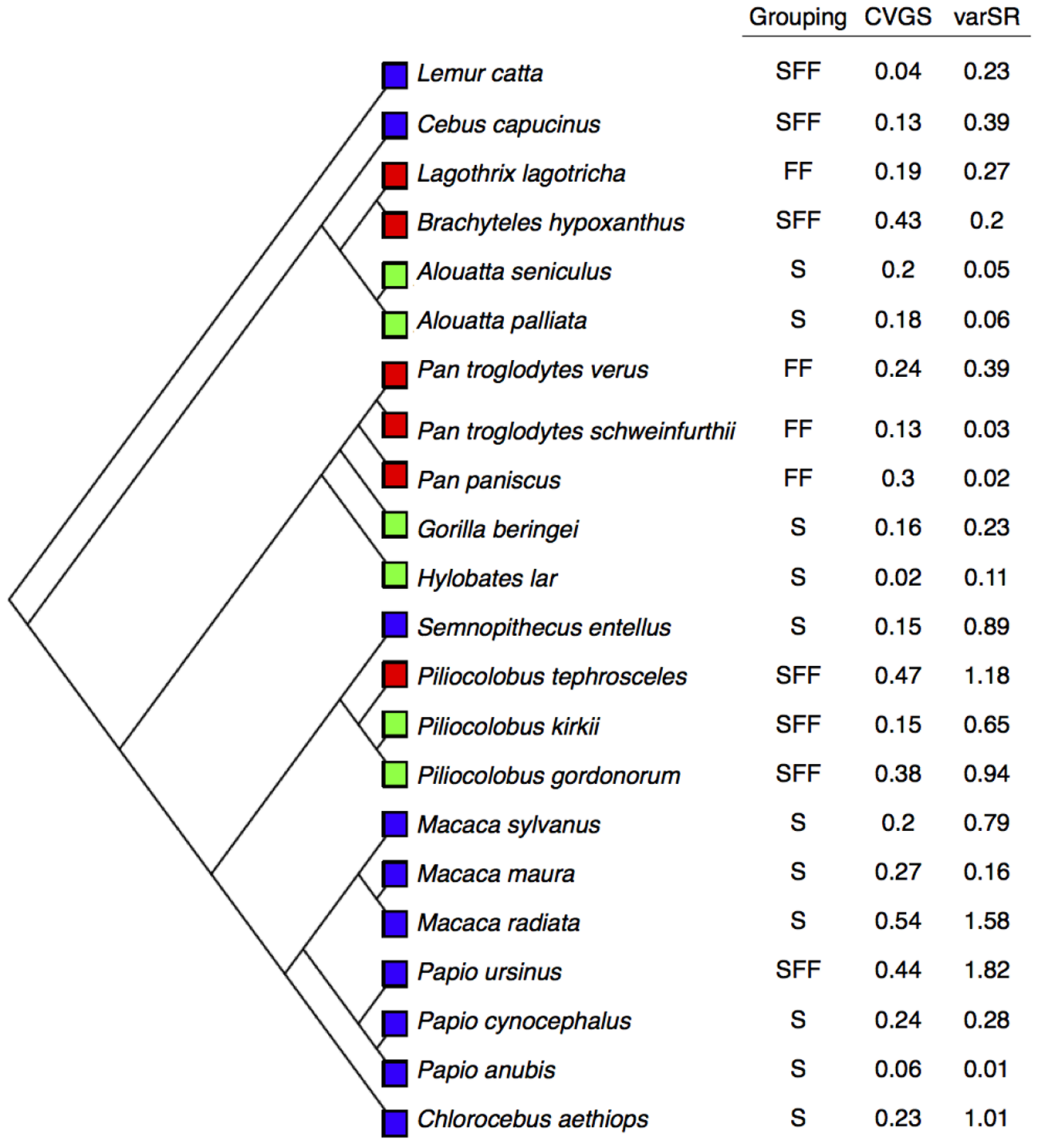
Distribution of Dispersal regimes (blue: Male-biased; red: Female-biased; green: Bi-sexual) among the 22 primate species in our study. Grouping (S = stable; FF = fission-fusion; SFF = sometimes fission-fusion), CV in Group size (CVGS), and variation in Sex ratio (varSR = 100*variance in arcsine square-root transformed sex ratio; see Table S1).

Dispersal among species represented in our sample exhibit a strong phylogenetic signal (LRT, χ^2^_0_+χ^2^_1_ = 12.9, P = 0.00017; Figure 1), consistent with previous studies based on different primate species [18], [27] and with our assumption that dispersal represents a basal and evolved constraining influence on social behavior [28]. By contrast, although variants in Grouping (SFF) occurred under all three dispersal regimes, S was only found under male or bi-sexual dispersal (i.e., when males disperse) and FF was only found under female dispersal (Figure 1). There was no residual phylogenetic signal in Grouping patterns after accounting for Dispersal regime, consistent with our predictions for responding traits (Table 1).

**Table 1:** Binary phylogenetic regression of Grouping on Dispersal regime.

Dependent variable	Coefficient	Estimate	95% Conf. Interval	P-value
[S or SFF] vs. FF	Female	1.80	(0.72, 3.53)	0.0034
	Male vs. Female	−2.26	(−4.62, −1.07)	0.0023
	Both vs. Female	−1.68	(−4.03, −0.031)	0.010
	α (phylogenetic signal)	0.10		0.13
S vs. [FF or SFF]	Female	−2.02	(−2.72, −0.55)	0.008
	Male vs. Female	2.69	(0.60, 4.47)	0.014
	Both vs. Female	2.85	(0.65, 5.17)	0.010
	α (phylogenetic signal)	0.06		0.39

Grouping was treated as a binary variable by either combining S and SFF, or combining FF and SFF. The independent variables were coded to give the coefficient for Female, and then the contrasts between Male and Female, and between Both and Female. The parameter α gives the strength of phylogenetic signal.

**Figure 2:**
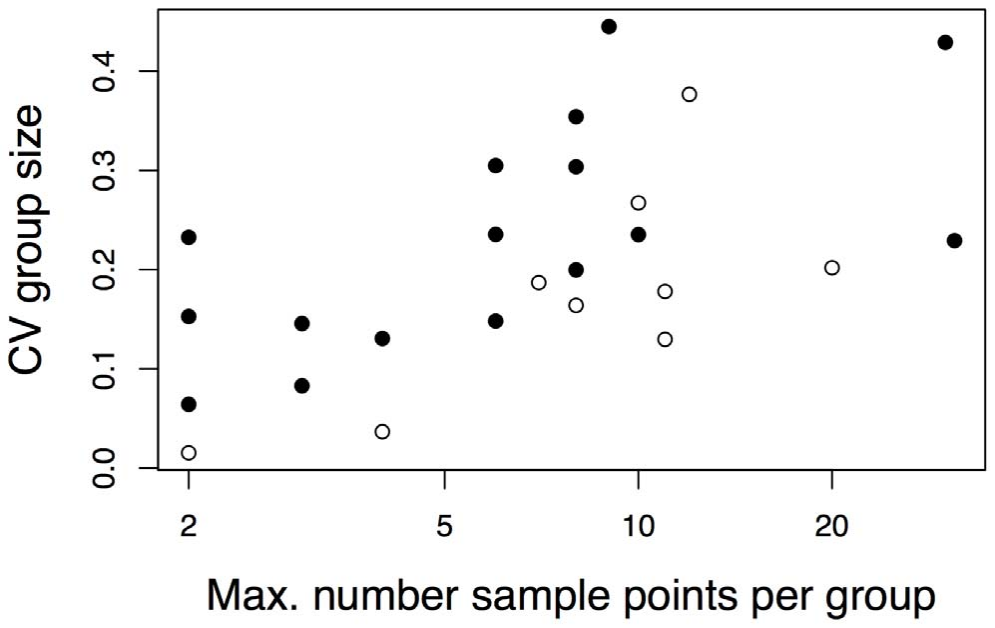
For longitudinal studies, the relationship between the coefficient of variation (CV) in Group size through time versus the maximum number of samples taken for any group within the study (note the log scale). For longitudinal group-level studies (solid circles) in which more than one group was followed through time, the CV was calculated as the mean CV in group size among groups, and the number of samples corresponds to the group with the greatest number of observations. For longitudinal population-level studies (open circles) the CV was calculated for the mean group size through time, and the number of samples equals the number of observations.

When quantifying variation in group size through time in the 25 longitudinal population and group studies, the coefficient of variation (CV) in group size increased with the number of data points over which the group size was measured (Figure 2). In other words, the greater the number of observations during the study, the greater the variation observed. Therefore, to remove this bias in the estimates of variation in group size, we regressed CV in group size against the maximum number of observations made per group within a study and used the residual variation as a comparative metric of variation in group size. In contrast to group size, the variation in adult sex ratio was independent of both the number of observations and duration of the studies.

**Figure 3:**
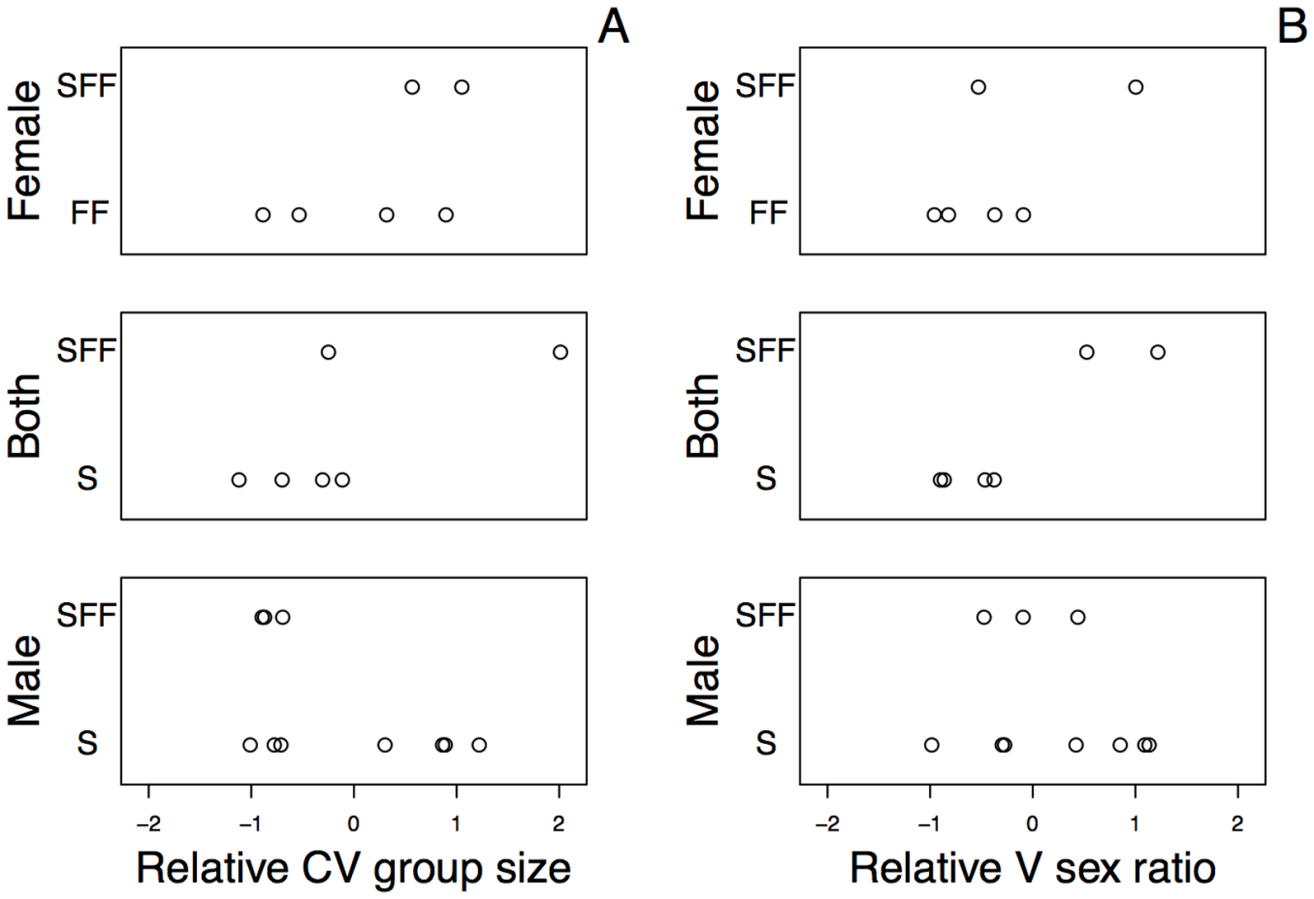
Grouping traits (S, FF, and SFF) for species divided by Dispersal regime (Male-biased, Female-biased, or Bi-sexual) versus (A) the relative CV in Group size and (B) the relative variance in sex ratio. For longitudinal studies, the relative CV in group sizes were calculated as the residuals of a regression of CV in group size against the log_10_ maximum number of samples per group (Figure 2), where the intercepts were allowed to differ. Similarly, for cross-sectional studies the mean CV in group size was subtracted from the CVs from each study. The residuals for all three types of studies (longitudinal group, longitudinal population, and cross-sectional) were then standardized to have a variance of one. This standardization makes the variation in CVs in group size the same for all three types of study. The analysis of variance in sex ratio (after arcsine-square-root transformation) was performed similarly to make the variation within the three types of studies the same.

Dispersal regime was not related to variation in group size or variation in adult sex ratio (Figure 1). However, Dispersal regime did appear to constrain whether Grouping patterns were responding to demographic variation (bi-sexual and female dispersal) or not (male dispersal; Figure 3). Compared to species with male dispersal, species with bi-sexual or female dispersal were relatively more likely to show SFF Grouping if they experienced greater variation in group size or adult sex ratios. In other words, in phylogenetic logistic regressions of whether or not species showed SFF, there was a statistically significant interaction between Dispersal (Male vs. Female+Bi-sexual) and variation in group size (P = 0.022, Table 2), and a marginally significant interaction for variation in sex ratio (P = 0.052, Table 2). Therefore, whether or not variation in group size and adult sex ratios affected the species' grouping patterns depended on Dispersal regime. The absence of an effect of variation in group size and sex ratio under male dispersal implies that male dispersal (probably driven by its corollary of female philopatry) overrides the influence of demographic variation on grouping dynamics.

**Table 2:** Binary phylogenetic regressions of Grouping (SFF vs. not SFF) on Dispersal regime and variation in group size, and on Dispersal regime and variation in adult sex ratio.

Dependent variable	Coefficient	Estimate	95% Conf. Interval	P-value
SFF vs. [S or FF]	variation in group size	−1.24	(−2.56, 0.35)	0.18
	[Female or Bi] vs. Male	0.16	(−2.13, 1.92)	0.96
	[V in GS]*[[F or B] vs. M]	2.56	(0.30, 5.97)	0.022
	α (phylogenetic signal)	0.046		0.52
SFF vs. [S or FF]	variation in sex ratio	−0.54	(−3.00, 0.68)	0.57
	[Female or Bi] vs. Male	0.29	(−1.80. 2.95)	0.81
	[V in SR]*[[F or B] vs. M]	2.83	(−0.05, 7.24)	0.052
	α (phylogenetic signal)	0		1.0

Grouping was treated as a binary variable combining S and FF to compare with SFF. Variation in group size was measured as the residual CV in group size after removing the effect of length of the data set (Figure 2). Variation in sex ratio was measured as the variance in arcsine square-root transformed adult sex ratio. Female and bi-sexual Dispersal were combined and compared to male Dispersal. The parameter α gives the strength of phylogenetic signal.

## Discussion

Our rationale for treating Dispersal as a constraining trait was predicated on the assumptions that it is a relatively evolutionarily conservative trait with a strong phylogenetic signal and that, compared to a responding trait, dispersal would exhibit little intraspecific variation. The first of these assumptions has been demonstrated in other more comprehensive comparative phylogenetic analyses [11], [18], [25], [28], and confirmed here with our more limited but longitudinal sample of species. The second of these assumptions would require more comparative long-term data to quantify the conditions under which deviations from normative dispersal patterns in a number of species occur [29]. Analyses of the extent and distribution of variation in normative dispersal regimes might be expected to reveal that even such a conservative behavioral trait as dispersal can respond to extreme demographic conditions, and thus shift from being a constraining to a responding trait in relatively short spans of time. We do not yet have the data needed to assess the degree to which other constraining traits (e.g., life histories) or demographic conditions might affect dispersal per se and its constraining properties.

We found no evidence that Dispersal constrains demographic variation directly (Figure 3). Indeed, traits such as group size and adult sex ratio fluctuate widely and unpredictably within species and over time, emphasizing the need for cautious application interpretations of species norms in comparative studies [22]. Furthermore, our finding that demographic variation increases with the number of data points highlights the importance of long-term data to quantify the demographic variables underlying complex social behavior, especially in long-lived animals such as primates.

In contrast to Dispersal, Grouping was responsive to demographic variation under female-biased and bi-sexual dispersal regimes, with the variant form of grouping, SFF, occurring when group size and adult sex ratio were highly variable. Assuming that SFF reflects the ability to adjust grouping patterns from the invariant forms of grouping, FF or S, that occur with lower demographic variation under female or bi-sexual dispersal, respectively, then the question remains, why was there no effect of demographic variation under male dispersal?

We suggest that in species with male dispersal, the access philopatric females have to extended female kin relationships may mitigate against the unpredictable social strains arising from fluctuating demographic conditions. Extended female kin relationships have been shown to buffer females from intra- and intersexual conflicts [44]; that these relationships might also permit more stable grouping patterns would represent an additional advantage. By contrast, under female or bi-sexual dispersal, females lack access to extended kin, and therefore must rely on adjustments in their grouping patterns to negotiate fluctuating social relationships. This hypothesis explains why the extended female kin groups that become established under male dispersal appear to be more resilient (and therefore less responsive) to demographic variation than the groups of unrelated females that typically occur under female or bi-sexual dispersal.

It is possible that our findings reflect more about the nature of our sample species than about the effects of demographic variation on different kinds of behavioral traits. We limited our dataset to published studies that provided systematic data on group size and adult sex ratios; these obviously do not represent all long-term primate studies. Moreover, our dataset does not permit us to evaluate alternative hypotheses about the ways in which behavior patterns other than grouping might respond to demographic variation. For example, females living in extended kin groups under male dispersal might respond to high demographic variation by adjusting their social alliances, or their rates or types of interactions with one another instead of their Grouping *per se* (e.g., [45]). Alternatively, male dispersal regimes may permit males to make finer-grain adjustments in group sizes and sex ratios through secondary dispersal with more frequent movements across a larger number of groups than occurs for males or females under other dispersal regimes (e.g., [46], [47]). Separating these alternatives would require more complete information on the comparative demographic variation of populations, instead of just one or a subset of groups that comprise most of the studies in our dataset. Indeed, we hope that the potential value of our approach of incorporating intraspecific variation in demographic variables into models of behavioral evolution will stimulate greater access to the raw data needed for these kinds of analyses.

Almost all of our study species have experienced recent habitat and population disturbances as a result of anthropogenic activities. This makes it difficult to evaluate how well our measurements of variation in group size and adult sex ratio, and our characterization of grouping patterns, represent historical, species-specific norms. Nonetheless, our demonstration of the responsiveness of grouping patterns to extreme demographic variation under some dispersal regimes provides insights into how major demographic changes might have led to evolutionary shifts in social states and to why some social states (e.g., female kin groups) might be more resilient to change (sensu [48], [49]).

By distinguishing between constraining and responding traits, our approach permits us to begin to disentangle the complex interactions between demographic variation and behavior, highlighting the conditions under which demography can drive behavioral flexibility. Recent investigations have shown the importance of behavioral flexibility to thermoregulation, and thus both the potential of primates to adjust to projected increases in global temperatures and the energetic limitations to these behavioral adjustments [50]–[53]. Brain size has also been shown to be positively related to the flexibility of primate social organizations and the adaptive potential that this flexibility affords to changing environmental conditions [22].

Primates are unique study subjects for exploring the interactions between constraining and responding behavioral traits and demographic variation because they can experience both high levels of demographic variation and a wide range of social opportunities during their long lives. Understanding these interactions will help predict which species will be more or less able to react quickly and facultatively to demographic fluctuations in increasingly human-dominated ecosystems and landscapes [54] as well as under the impacts of climate change on their habitats [50]–[52].

## Supporting Information

Table S1 **Summary data on demographic and behavioral variation.** Type: longgroup = longitudinal group, longpop = longitudinal population, crosspop = cross population; Grouping: S = stable, FF = fission-fusion, SFF = sometimes fission-fusion; N Groups: average number of groups sampled per year; Mean Group size: mean size of groups among years; CV Group size: CVs calculated through time for each group; Mean Sex ratio: mean adult sex ratios (males/females) per group among years; V arcsinSquareRoot Sex ratio: variance in the arcsine-square-root transformed proportion of males (males/[males+females]) calculated through time for each group; Mean N obs: mean number of observations made per group or population; Max N obs: maximum number of observations made per group or population; Duration: years between the first and last observation of a group; Note that when there was more than one group in a longitudinal study, values were averaged among groups; See text for definitions. Sources: See Table S3.
(PDF)

Table S2 **Annual data on demographic and behavioral variation.** Type: longgroup = longitudinal group, longpop = longitudinal population, crosspop = cross population; Group identifier: for longgroup studies, an integer identifying separate groups, set to one for other studies; Year in study sequence: the year of the study, starting with year 1 for each group or population; Grouping: S = stable, FF = fission-fusion, SFF = sometimes fission-fusion; Ad males and Ad females: numbers of adult males and females per group; Sex ratio: Ad males divided by Ad females; Group size: for longpop studies, this is the average group size in a given year; See text for definitions. Sources: See Table S3.
(PDF)

Table S3 **References to Table S1 and Table S2.**
(PDF)
