# Supplementary material for: Behavioral Flexibility and the Evolution of Primate Social States
Source: PLoS One. 2014 Dec 3;9(12):e114099. doi: 10.1371/journal.pone.0114099 (PMC4254976; doi:10.1371/journal.pone.0114099)
Supplement: Table S1 — Summary data on demographic and behavioral variation. Type: longgroup = longitudinal group, longpop = longitudinal population, crosspop = cross population; Grouping: S = stable, FF = fission-fusion, SFF = sometimes fission-fusion; N Groups: average number of groups sampled per year; Mean Group size: mean size of groups among years; CV Group size: CVs calculated through time for each group; Mean Sex ratio: mean adult sex ratios (males/females) per group among years; V arcsinSquareRoot Sex ratio: variance in the arcsine-square-root transformed proportion of males (males/[males+females]) calculated through time for each group; Mean N obs: mean number of observations made per group or population; Max N obs: maximum number of observations made per group or population; Duration: years between the first and last observation of a group; Note that when there was more than one group in a longitudinal study, values were averaged among groups; See text for definitions. Sources: See Table S3. (PDF) [file pone.0114099.s001.pdf]

Table S1: Summary data on demographic and behavioral variation.

Source	Taxa	Type	Dispersal	Grouping	N groups	Mean Group size	CV Group size	Mean Sex ratio	V arcsinSquareRoot Sex ratio	Mean N obs	Max N obs	Duration (yrs)
[1]	*Alouatta palliata*	longpop	Both	S	27.36	13.71	0.178	0.594	0.00063	11	11	25
[2]	*Alouatta seniculus*	longpop	Both	S	28.55	4.57	0.202	0.616	0.00047	20	20	24
[3]	*Brachyteles hypoxanthus*	longgroup	Female	SFF	1.00	59.40	0.429	0.731	0.00204	30	30	30
[1]	*Cebus capucinus*	longpop	Male	SFF	23.73	15.50	0.130	0.656	0.00388	11	11	25
[4, 5]	*Chlorocebus aethiops*	longgroup	Male	S	5.00	20.81	0.233	0.630	0.01011	2	2	9
[6-9]	*Chlorocebus aethiops*	longgroup	Male	S	3.00	17.56	0.354	0.595	0.00806	7.7	8	9.3
[10]	*Gorilla beringei*	longpop	Both	S	31.00	9.80	0.164	0.531	0.00231	8	8	40
[11]	*Hylobates lar*	longpop	Both	S	12.42	4.59	0.015	0.794	0.00109	2	2	12
[11]	*Hylobates lar*	longgroup	Both	S	5.00	4.10	0.229	0.768	0.00428	22	31	22
[12, 13]	*Lagothrix lagotricha*	longpop	Female	FF	11.00	17.86	0.187	0.598	0.00271	7	7	7
[14]	*Lemur catta*	longpop	Male	SFF	8.50	11.45	0.037	0.718	0.00227	4	4	15
[15]	*Macaca maura*	longpop	Male	S	3.20	30.30	0.267	0.533	0.00607	10	10	11
[16]	*Macaca radiata*	crossgroup	Male	S	21.00	15.29	0.540	0.609	0.01585	21	21	1
[16]	*Macaca radiata*	longgroup	Male	S	4.00	23.10	0.153	0.487	0.00063	1.6	2	3.2
[17]	*Macaca sylvanus*	longgroup	Male	S	5.00	40.32	0.200	0.710	0.00789	4	8	4
[18, 19]	*Pan paniscus*	longgroup	Female	FF	1.00	59.83	0.305	0.711	0.00022	6	6	8
[20]	*Pan troglodytes schweinfurthii*	longgroup	Female	FF	1.00	48.00	0.131	0.649	0.00027	4	4	20
[21]	*Pan troglodytes schweinfurthii*	longgroup	Female	FF	1.00	150.67	0.083	0.622	0.00134	3	3	12
[22]	*Pan troglodytes verus*	longgroup	Female	FF	1.00	32.40	0.235	0.404	0.00390	10	10	10
[23, 24]	*Papio anubis*	longgroup	Male	S	1.00	110.00	0.064	0.536	0.00011	2	2	7
[24-27]	*Papio cynocephalus*	longgroup	Male	S	1.00	38.33	0.235	0.587	0.00276	6	6	17
[24-28]	*Papio cynocephalus*	longgroup	Male	S	1.00	47.13	0.304	0.586	0.00328	8	8	17
[29]	*Papio ursinus*	crossgroup	Male	SFF	4.00	29.25	0.439	0.482	0.01820	4	4	1
[30]	*Papio ursinus*	crossgroup	Male	SFF	4.00	34.25	0.442	0.414	0.01419	4	4	1
[31]	*Piliocolobus gordonorum*	longpop	Both	SFF	1.00	28.42	0.377	0.459	0.00945	7.7	12	7.3
[32]	*Piliocolobus kirkii*	longgroup	Both	SFF	11.00	17.62	0.146	0.510	0.00651	2.7	3	2.7
[33]	*Piliocolobus tephrosceles*	crossgroup	Female	SFF	2.00	36.00	0.471	0.589	0.01177	2	2	1
[34]	*Piliocolobus tephrosceles*	longgroup	Female	SFF	3.00	23.65	0.445	0.611	0.01785	4.7	9	9
[35, 36]	*Semnopithecus entellus*	longgroup	Male	S	10.00	18.31	0.148	0.517	0.00891	5.2	6	5.2

Legend: Type: longgroup=longitudinal group, longpop=longitudinal population, crosspop=cross population; **Grouping:** S=stable, FF=fission-fusion, SFF=sometimes fission-fusion; **N Groups:** average number of groups sampled per year; **Mean Group size:** mean size of groups among years; **CV Group size:** CVs calculated through time for each group; **Mean Sex ratio:** mean adult sex ratios (males/females) per group among years; **V arcsinSquareRoot Sex ratio:** variance in the arcsine-square-root transformed proportion of males (males/[males+females]) calculated through time for each group; **Mean N obs:** mean number of observations made per group or population; **Max N obs:** maximum number of observations made per group or population; **Duration:** years between the first and last observation of a group; Note that when there was more than one group in a longitudinal study, values were averaged among groups; See text for definitions.

Sources: See Table S3.
