# Supplementary material for: Behavioral Flexibility and the Evolution of Primate Social States
Source: PLoS One. 2014 Dec 3;9(12):e114099. doi: 10.1371/journal.pone.0114099 (PMC4254976; doi:10.1371/journal.pone.0114099)
Supplement: Table S2 — Annual data on demographic and behavioral variation. Type: longgroup = longitudinal group, longpop = longitudinal population, crosspop = cross population; Group identifier: for longgroup studies, an integer identifying separate groups, set to one for other studies; Year in study sequence: the year of the study, starting with year 1 for each group or population; Grouping: S = stable, FF = fission-fusion, SFF = sometimes fission-fusion; Ad males and Ad females: numbers of adult males and females per group; Sex ratio: Ad males divided by Ad females; Group size: for longpop studies, this is the average group size in a given year; See text for definitions. Sources: See Table S3. (PDF) [file pone.0114099.s002.pdf]

Table S2: Annual data on demographic and behavioral variation.

Source	Taxa	Type	Group identifier	Year	Year in study sequence	Dispersal	Grouping	Ad males	Ad females	Sex ratio	N groups	Group size
[1]	*Alouatta palliata*	longpop	1	1983	1	Both	S	NA	NA	0.390	19	11.40
[1]	*Alouatta palliata*	longpop	1	1984	2	Both	S	NA	NA	0.550	23	12.80
[1]	*Alouatta palliata*	longpop	1	1985	3	Both	S	NA	NA	0.620	19	13.80
[1]	*Alouatta palliata*	longpop	1	1986	4	Both	S	NA	NA	0.510	19	16.60
[1]	*Alouatta palliata*	longpop	1	1987	5	Both	S	NA	NA	0.570	16	11.30
[1]	*Alouatta palliata*	longpop	1	1988	6	Both	S	NA	NA	0.540	12	17.70
[1]	*Alouatta palliata*	longpop	1	1990	7	Both	S	NA	NA	0.550	27	16.00
[1]	*Alouatta palliata*	longpop	1	1992	9	Both	S	NA	NA	0.560	35	16.10
[1]	*Alouatta palliata*	longpop	1	1999	16	Both	S	NA	NA	0.560	46	11.80
[1]	*Alouatta palliata*	longpop	1	2003	20	Both	S	NA	NA	0.460	44	12.00
[1]	*Alouatta palliata*	longpop	1	2007	24	Both	S	NA	NA	0.530	41	11.30
[2]	*Alouatta seniculus*	longpop	1	1976	1	Both	S	33	56	0.590	21	5.05
[2]	*Alouatta seniculus*	longpop	1	1977	2	Both	S	30	55	0.550	21	4.81
[2]	*Alouatta seniculus*	longpop	1	1978	3	Both	S	36	55	0.650	22	4.73
[2]	*Alouatta seniculus*	longpop	1	1979	4	Both	S	37	62	0.600	24	5.04
[2]	*Alouatta seniculus*	longpop	1	1981	6	Both	S	42	76	0.550	28	5.11
[2]	*Alouatta seniculus*	longpop	1	1984	9	Both	S	42	78	0.540	30	4.80
[2]	*Alouatta seniculus*	longpop	1	1985	10	Both	S	59	86	0.690	33	5.06
[2]	*Alouatta seniculus*	longpop	1	1986	11	Both	S	61	90	0.680	33	5.03
[2]	*Alouatta seniculus*	longpop	1	1987	12	Both	S	55	98	0.560	34	5.03
[2]	*Alouatta seniculus*	longpop	1	1988	13	Both	S	55	97	0.570	34	4.88
[2]	*Alouatta seniculus*	longpop	1	1989	14	Both	S	58	106	0.550	36	5.08
[2]	*Alouatta seniculus*	longpop	1	1990	15	Both	S	59	106	0.560	36	5.44
[2]	*Alouatta seniculus*	longpop	1	1991	16	Both	S	62	110	0.560	36	5.47
[2]	*Alouatta seniculus*	longpop	1	1992	17	Both	S	57	106	0.540	36	5.06
[2]	*Alouatta seniculus*	longpop	1	1993	18	Both	S	65	89	0.730	33	5.36
[2]	*Alouatta seniculus*	longpop	1	1994	19	Both	S	26	44	0.590	29	2.86
[2]	*Alouatta seniculus*	longpop	1	1995	20	Both	S	26	39	0.670	29	2.76
[2]	*Alouatta seniculus*	longpop	1	1996	21	Both	S	29	45	0.640	23	3.48
[2]	*Alouatta seniculus*	longpop	1	1997	22	Both	S	16	33	0.480	18	2.78
[2]	*Alouatta seniculus*	longpop	1	1999	24	Both	S	16	30	0.530	15	3.47
[3]	*Brachyteles hypoxanthus*	longgroup	1	1982	1	Female	S	6	8	0.750	1	22.00
[3]	*Brachyteles hypoxanthus*	longgroup	1	1983	2	Female	S	8	8	1.000	1	23.00
[3]	*Brachyteles hypoxanthus*	longgroup	1	1984	3	Female	S	8	8	1.000	1	27.00
[3]	*Brachyteles hypoxanthus*	longgroup	1	1985	4	Female	S	8	9	0.890	1	30.00
[3]	*Brachyteles hypoxanthus*	longgroup	1	1986	5	Female	S	8	9	0.890	1	33.00

Source	Taxa	Type	Group identifier	Year	Year in study sequence	Dispersal	Grouping	Ad males	Ad females	Sex ratio	N groups	Group size
[3]	*Brachyteles hypoxanthus*	longgroup	1	1987	6	Female	SFF	10	9	1.110	1	33.00
[3]	*Brachyteles hypoxanthus*	longgroup	1	1988	7	Female	SFF	10	9	1.110	1	32.00
[3]	*Brachyteles hypoxanthus*	longgroup	1	1989	8	Female	SFF	8	11	0.730	1	37.00
[3]	*Brachyteles hypoxanthus*	longgroup	1	1990	9	Female	SFF	10	11	0.910	1	43.00
[3]	*Brachyteles hypoxanthus*	longgroup	1	1991	10	Female	SFF	10	13	0.770	1	41.00
[3]	*Brachyteles hypoxanthus*	longgroup	1	1992	11	Female	SFF	10	15	0.670	1	47.00
[3]	*Brachyteles hypoxanthus*	longgroup	1	1993	12	Female	SFF	11	15	0.730	1	50.00
[3]	*Brachyteles hypoxanthus*	longgroup	1	1994	13	Female	SFF	13	16	0.810	1	49.00
[3]	*Brachyteles hypoxanthus*	longgroup	1	1995	14	Female	SFF	13	16	0.810	1	55.00
[3]	*Brachyteles hypoxanthus*	longgroup	1	1996	15	Female	SFF	16	16	1.000	1	56.00
[3]	*Brachyteles hypoxanthus*	longgroup	1	1997	16	Female	FF	17	16	1.060	1	59.00
[3]	*Brachyteles hypoxanthus*	longgroup	1	1998	17	Female	FF	18	17	1.060	1	62.00
[3]	*Brachyteles hypoxanthus*	longgroup	1	1999	18	Female	FF	18	18	1.000	1	62.00
[3]	*Brachyteles hypoxanthus*	longgroup	1	2000	19	Female	FF	23	19	1.210	1	64.00
[3]	*Brachyteles hypoxanthus*	longgroup	1	2001	20	Female	FF	25	19	1.320	1	68.00
[3]	*Brachyteles hypoxanthus*	longgroup	1	2002	21	Female	FF	26	20	1.300	1	66.00
[3]	*Brachyteles hypoxanthus*	longgroup	1	2003	22	Female	FF	28	22	1.270	1	78.00
[3]	*Brachyteles hypoxanthus*	longgroup	1	2004	23	Female	FF	29	22	1.320	1	82.00
[3]	*Brachyteles hypoxanthus*	longgroup	1	2005	24	Female	FF	31	22	1.410	1	83.00
[3]	*Brachyteles hypoxanthus*	longgroup	1	2006	25	Female	FF	33	24	1.380	1	82.00
[3]	*Brachyteles hypoxanthus*	longgroup	1	2007	26	Female	FF	36	27	1.330	1	83.00
[3]	*Brachyteles hypoxanthus*	longgroup	1	2008	27	Female	FF	37	26	1.420	1	99.00
[3]	*Brachyteles hypoxanthus*	longgroup	1	2009	28	Female	FF	37	27	1.370	1	102.00
[3]	*Brachyteles hypoxanthus*	longgroup	1	2010	29	Female	FF	36	31	1.160	1	107.00
[3]	*Brachyteles hypoxanthus*	longgroup	1	2011	30	Female	FF	35	31	1.130	1	107.00
[1]	*Cebus capucinus*	longpop	1	1983	1	Male	SFF	2	NA	0.470	20	11.50
[1]	*Cebus capucinus*	longpop	1	1984	2	Male	SFF	2.3	NA	0.470	25	13.60
[1]	*Cebus capucinus*	longpop	1	1985	3	Male	SFF	2.6	NA	0.510	13	14.80
[1]	*Cebus capucinus*	longpop	1	1986	4	Male	SFF	3.3	NA	0.610	18	16.40
[1]	*Cebus capucinus*	longpop	1	1987	5	Male	SFF	3.7	NA	0.830	10	16.70
[1]	*Cebus capucinus*	longpop	1	1988	6	Male	SFF	3.7	NA	0.850	8	16.40
[1]	*Cebus capucinus*	longpop	1	1990	8	Male	SFF	4.4	NA	0.830	18	17.70
[1]	*Cebus capucinus*	longpop	1	1992	10	Male	SFF	4	NA	0.780	30	18.00
[1]	*Cebus capucinus*	longpop	1	1999	18	Male	SFF	NA	NA	0.690	31	16.80
[1]	*Cebus capucinus*	longpop	1	2003	22	Male	SFF	NA	NA	1.120	49	13.40
[1]	*Cebus capucinus*	longpop	1	2007	26	Male	SFF	NA	NA	1.080	39	15.20
[4, 5]	*Chlorocebus aethiops*	longgroup	1	1963	1	Male	S	2	2	1.000	1	14.00
[4, 5]	*Chlorocebus aethiops*	longgroup	1	1971	8	Male	S	2	4	0.500	1	10.00
[4, 5]	*Chlorocebus aethiops*	longgroup	2	1963	1	Male	S	1.8	3.15	0.510	1	15.95

Source	Taxa	Type	Group identifier	Year	Year in study sequence	Dispersal	Grouping	Ad males	Ad females	Sex ratio	N groups	Group size
[4, 5]	*Chlorocebus aethiops*	longgroup	2	1971	8	Male	S	4	5	0.800	1	19.00
[4, 5]	*Chlorocebus aethiops*	longgroup	3	1963	1	Male	S	2.4	4	0.600	1	16.96
[4, 5]	*Chlorocebus aethiops*	longgroup	3	1971	8	Male	S	3	4	0.750	1	13.00
[4, 5]	*Chlorocebus aethiops*	longgroup	4	1963	1	Male	S	3	8.29	0.360	1	28.00
[4, 5]	*Chlorocebus aethiops*	longgroup	4	1971	8	Male	S	5	6	0.830	1	20.00
[4, 5]	*Chlorocebus aethiops*	longgroup	5	1963	1	Male	S	3.46	9.72	0.360	1	45.19
[4, 5]	*Chlorocebus aethiops*	longgroup	5	1971	8	Male	S	4	4	1.000	1	26.00
[6,7]	*Chlorocebus aethiops*	longgroup	1	1978	1	Male	S	7	8	0.875	1	30.00
[6,7]	*Chlorocebus aethiops*	longgroup	1	1979	2	Male	S	3	8	0.375	1	23.00
[6,7]	*Chlorocebus aethiops*	longgroup	1	1980	3	Male	S	3	4	0.750	1	28.00
[6,7]	*Chlorocebus aethiops*	longgroup	1	1982	4	Male	S	2	4	0.500	1	11.00
[6,7]	*Chlorocebus aethiops*	longgroup	1	1983	5	Male	S	3	3	1.000	1	14.00
[8]	*Chlorocebus aethiops*	longgroup	1	1984	6	Male	S	3	3	1.000	1	10.00
[8]	*Chlorocebus aethiops*	longgroup	1	1985	7	Male	S	1	3	0.333	1	10.00
[6,7]	*Chlorocebus aethiops*	longgroup	2	1978	1	Male	S	2	7	0.286	1	17.00
[6,7]	*Chlorocebus aethiops*	longgroup	2	1979	2	Male	S	2	7	0.286	1	19.00
[6,7]	*Chlorocebus aethiops*	longgroup	2	1980	3	Male	S	1	7	0.143	1	21.00
[6,7]	*Chlorocebus aethiops*	longgroup	2	1982	4	Male	S	2	6	0.333	1	18.00
[6,7]	*Chlorocebus aethiops*	longgroup	2	1983	5	Male	S	4	7	0.571	1	19.00
[6,7]	*Chlorocebus aethiops*	longgroup	2	1984	6	Male	S	4	7	0.571	1	21.00
[6,7]	*Chlorocebus aethiops*	longgroup	2	1985	7	Male	S	3	7	0.429	1	24.00
[9]	*Chlorocebus aethiops*	longgroup	2	1987	8	Male	S	4	4	1.000	1	13.00
[6,7]	*Chlorocebus aethiops*	longgroup	3	1978	1	Male	S	4	8	0.500	1	27.00
[6,7]	*Chlorocebus aethiops*	longgroup	3	1979	2	Male	S	4	8	0.500	1	23.00
[6,7]	*Chlorocebus aethiops*	longgroup	3	1980	3	Male	S	3	5	0.600	1	17.00
[6,7]	*Chlorocebus aethiops*	longgroup	3	1982	4	Male	S	2	4	0.500	1	12.00
[6,7]	*Chlorocebus aethiops*	longgroup	3	1983	5	Male	S	2	4	0.500	1	13.00
[6,7]	*Chlorocebus aethiops*	longgroup	3	1984	6	Male	S	2	4	0.500	1	12.50
[6,7]	*Chlorocebus aethiops*	longgroup	3	1985	7	Male	S	3	4	0.750	1	13.00
[9]	*Chlorocebus aethiops*	longgroup	3	1987	8	Male	S	2	2	1.000	1	8.00
[10]	*Gorilla beringei*	longpop	1	1971	1	Both	S	1	3	0.330	31	7.90
[10]	*Gorilla beringei*	longpop	1	1976	6	Both	S	2	6	0.330	28	8.80
[10]	*Gorilla beringei*	longpop	1	1981	10	Both	S	2	6	0.330	28	8.50
[10]	*Gorilla beringei*	longpop	1	1986	15	Both	S	2	6	0.330	29	9.20
[10]	*Gorilla beringei*	longpop	1	1989	18	Both	S	2.1	5.9	0.360	32	9.20
[10]	*Gorilla beringei*	longpop	1	2000	30	Both	S	3.06	5.24	0.590	32	10.90
[10]	*Gorilla beringei*	longpop	1	2003	30	Both	S	NA	NA	NA	32	11.40
[10]	*Gorilla beringei*	longpop	1	2010	30	Both	S	NA	3.54	0.500	36	12.50
[11]	*Hylobates lar*	longpop	1	1999	1	Both	S	12.49	12.49	1.000	12	4.54

Source	Taxa	Type	Group identifier	Year	Year in study sequence	Dispersal	Grouping	Ad males	Ad females	Sex ratio	N groups	Group size
[11]	*Hylobates lar*	longpop	1	2000	2	Both	S	13.51	11.99	1.130	12	NA
[11]	*Hylobates lar*	longpop	1	2001	3	Both	S	14	11	1.270	12	NA
[11]	*Hylobates lar*	longpop	1	2002	4	Both	S	15	10.5	1.430	12	NA
[11]	*Hylobates lar*	longpop	1	2003	5	Both	S	15.6	10.2	1.530	12	NA
[11]	*Hylobates lar*	longpop	1	2004	6	Both	S	16.37	9.82	1.670	12	NA
[11]	*Hylobates lar*	longpop	1	2005	7	Both	S	16	10	1.600	12	NA
[11]	*Hylobates lar*	longpop	1	2006	8	Both	S	16	10	1.600	12	NA
[11]	*Hylobates lar*	longpop	1	2007	9	Both	S	15	10.5	1.430	12	NA
[11]	*Hylobates lar*	longpop	1	2008	10	Both	S	15	10.5	1.430	12	NA
[11]	*Hylobates lar*	longpop	1	2009	11	Both	S	16.98	11.49	1.480	14	NA
[11]	*Hylobates lar*	longpop	1	2010	12	Both	S	19.98	10.49	1.900	15	4.64
[11]	*Hylobates lar*	longgroup	1	1978	1	Both	S	1	1	1.000	1	4.00
[11]	*Hylobates lar*	longgroup	1	1979	2	Both	S	1	1	1.000	1	4.00
[11]	*Hylobates lar*	longgroup	1	1980	3	Both	S	1	1	1.000	1	4.00
[11]	*Hylobates lar*	longgroup	1	1981	4	Both	S	1	1	1.000	1	4.00
[11]	*Hylobates lar*	longgroup	1	1982	5	Both	S	1	1	1.000	1	5.00
[11]	*Hylobates lar*	longgroup	1	1983	6	Both	S	2	1	2.000	1	5.00
[11]	*Hylobates lar*	longgroup	2	1980	1	Both	S	1	1	1.000	1	3.00
[11]	*Hylobates lar*	longgroup	2	1981	2	Both	S	2	1	2.000	1	4.00
[11]	*Hylobates lar*	longgroup	2	1982	3	Both	S	2	1	2.000	1	4.00
[11]	*Hylobates lar*	longgroup	2	1983	4	Both	S	1	1	1.000	1	3.00
[11]	*Hylobates lar*	longgroup	2	1984	5	Both	S	1	1	1.000	1	5.00
[11]	*Hylobates lar*	longgroup	2	1985	6	Both	S	1	1	1.000	1	6.00
[11]	*Hylobates lar*	longgroup	2	1986	7	Both	S	1	1	1.000	1	6.00
[11]	*Hylobates lar*	longgroup	2	1987	8	Both	S	2	1	2.000	1	6.00
[11]	*Hylobates lar*	longgroup	2	1988	9	Both	S	2	1	2.000	1	6.00
[11]	*Hylobates lar*	longgroup	2	1989	10	Both	S	2	1	2.000	1	6.00
[11]	*Hylobates lar*	longgroup	2	1990	11	Both	S	3	1	3.000	1	5.00
[11]	*Hylobates lar*	longgroup	2	1991	12	Both	S	2	1	2.000	1	5.00
[11]	*Hylobates lar*	longgroup	2	1992	13	Both	S	1	1	1.000	1	4.00
[11]	*Hylobates lar*	longgroup	2	1993	14	Both	S	1	1	1.000	1	4.00
[11]	*Hylobates lar*	longgroup	2	1994	15	Both	S	1	1	1.000	1	5.00
[11]	*Hylobates lar*	longgroup	2	1995	16	Both	S	1	1	1.000	1	5.00
[11]	*Hylobates lar*	longgroup	2	1996	17	Both	S	1	1	1.000	1	5.00
[11]	*Hylobates lar*	longgroup	2	1997	18	Both	S	2	1	2.000	1	5.00
[11]	*Hylobates lar*	longgroup	2	1998	19	Both	S	2	1	2.000	1	4.00
[11]	*Hylobates lar*	longgroup	2	1999	20	Both	S	2	1	2.000	1	4.00
[11]	*Hylobates lar*	longgroup	2	2000	21	Both	S	1	1	1.000	1	3.00
[11]	*Hylobates lar*	longgroup	2	2001	22	Both	S	2	1	2.000	1	4.00

Source	Taxa	Type	Group identifier	Year	Year in study sequence	Dispersal	Grouping	Ad males	Ad females	Sex ratio	N groups	Group size
[11]	*Hylobates lar*	longgroup	2	2002	23	Both	S	1	1	1.000	1	2.00
[11]	*Hylobates lar*	longgroup	2	2003	24	Both	S	1	1	1.000	1	2.00
[11]	*Hylobates lar*	longgroup	2	2004	25	Both	S	2	1	2.000	1	4.00
[11]	*Hylobates lar*	longgroup	2	2005	26	Both	S	2	1	2.000	1	5.00
[11]	*Hylobates lar*	longgroup	2	2006	27	Both	S	3	1	3.000	1	5.00
[11]	*Hylobates lar*	longgroup	2	2007	28	Both	S	3	1	3.000	1	5.00
[11]	*Hylobates lar*	longgroup	2	2008	29	Both	S	3	1	3.000	1	5.00
[11]	*Hylobates lar*	longgroup	2	2009	30	Both	S	3	1	3.000	1	4.00
[11]	*Hylobates lar*	longgroup	2	2010	31	Both	S	3	1	3.000	1	4.00
[11]	*Hylobates lar*	longgroup	3	1980	1	Both	S	1	1	1.000	1	2.00
[11]	*Hylobates lar*	longgroup	3	1981	2	Both	S	1	1	1.000	1	3.00
[11]	*Hylobates lar*	longgroup	3	1982	3	Both	S	1	1	1.000	1	4.00
[11]	*Hylobates lar*	longgroup	3	1983	4	Both	S	1	1	1.000	1	4.00
[11]	*Hylobates lar*	longgroup	3	1984	5	Both	S	1	1	1.000	1	5.00
[11]	*Hylobates lar*	longgroup	3	1985	6	Both	S	1	1	1.000	1	5.00
[11]	*Hylobates lar*	longgroup	3	1986	7	Both	S	1	1	1.000	1	4.00
[11]	*Hylobates lar*	longgroup	3	1987	8	Both	S	1	1	1.000	1	4.00
[11]	*Hylobates lar*	longgroup	3	1988	9	Both	S	1	1	1.000	1	5.00
[11]	*Hylobates lar*	longgroup	3	1989	10	Both	S	1.5	1.5	NA	1	5.00
[11]	*Hylobates lar*	longgroup	3	1990	11	Both	S	1	1	1.000	1	4.00
[11]	*Hylobates lar*	longgroup	3	1991	12	Both	S	1	1	1.000	1	4.00
[11]	*Hylobates lar*	longgroup	3	1992	13	Both	S	1	2	0.500	1	5.00
[11]	*Hylobates lar*	longgroup	3	1993	14	Both	S	1	1	1.000	1	4.00
[11]	*Hylobates lar*	longgroup	3	1994	15	Both	S	2	1	2.000	1	5.00
[11]	*Hylobates lar*	longgroup	3	1995	16	Both	S	2	1	2.000	1	5.00
[11]	*Hylobates lar*	longgroup	3	1996	17	Both	S	2	2	1.000	1	5.00
[11]	*Hylobates lar*	longgroup	3	1997	18	Both	S	2	2	1.000	1	4.00
[11]	*Hylobates lar*	longgroup	3	1998	19	Both	S	2	2	1.000	1	5.00
[11]	*Hylobates lar*	longgroup	3	1999	20	Both	S	1	1	1.000	1	3.00
[11]	*Hylobates lar*	longgroup	3	2000	21	Both	S	1	1	1.000	1	3.00
[11]	*Hylobates lar*	longgroup	3	2001	22	Both	S	1	1	1.000	1	3.00
[11]	*Hylobates lar*	longgroup	3	2002	23	Both	S	1	1	1.000	1	3.00
[11]	*Hylobates lar*	longgroup	3	2003	24	Both	S	1	1	1.000	1	3.00
[11]	*Hylobates lar*	longgroup	3	2004	25	Both	S	1	1	1.000	1	3.00
[11]	*Hylobates lar*	longgroup	3	2005	26	Both	S	1	1	1.000	1	3.00
[11]	*Hylobates lar*	longgroup	3	2006	27	Both	S	2	1	2.000	1	3.00
[11]	*Hylobates lar*	longgroup	3	2007	28	Both	S	2	1	2.000	1	3.00
[11]	*Hylobates lar*	longgroup	3	2008	29	Both	S	2	1	2.000	1	3.00
[11]	*Hylobates lar*	longgroup	3	2009	30	Both	S	1	1	1.000	1	2.00

Source	Taxa	Type	Group identifier	Year	Year in study sequence	Dispersal	Grouping	Ad males	Ad females	Sex ratio	N groups	Group size
[11]	*Hylobates lar*	longgroup	3	2010	31	Both	S	1	1	1.000	1	3.00
[11]	*Hylobates lar*	longgroup	4	1981	1	Both	S	1	1	1.000	1	2.00
[11]	*Hylobates lar*	longgroup	4	1982	2	Both	S	1	1	1.000	1	2.00
[11]	*Hylobates lar*	longgroup	4	1983	3	Both	S	1	1	1.000	1	2.00
[11]	*Hylobates lar*	longgroup	4	1984	4	Both	S	1	1	1.000	1	3.00
[11]	*Hylobates lar*	longgroup	4	1985	5	Both	S	1	1	1.000	1	3.00
[11]	*Hylobates lar*	longgroup	4	1986	6	Both	S	1	1	1.000	1	3.00
[11]	*Hylobates lar*	longgroup	4	1987	7	Both	S	2	1	2.000	1	4.00
[11]	*Hylobates lar*	longgroup	4	1988	8	Both	S	2	1	2.000	1	5.00
[11]	*Hylobates lar*	longgroup	4	1989	9	Both	S	2	1	2.000	1	5.00
[11]	*Hylobates lar*	longgroup	4	1990	10	Both	S	2	1	2.000	1	5.00
[11]	*Hylobates lar*	longgroup	4	1991	11	Both	S	2	1	2.000	1	6.00
[11]	*Hylobates lar*	longgroup	4	1992	12	Both	S	2	1	2.000	1	6.00
[11]	*Hylobates lar*	longgroup	4	1993	13	Both	S	3	1	3.000	1	6.00
[11]	*Hylobates lar*	longgroup	4	1994	14	Both	S	1	1	1.000	1	5.00
[11]	*Hylobates lar*	longgroup	4	1995	15	Both	S	1	1	1.000	1	5.00
[11]	*Hylobates lar*	longgroup	4	1996	16	Both	S	2	1	2.000	1	5.00
[11]	*Hylobates lar*	longgroup	4	1997	17	Both	S	2	1	2.000	1	5.00
[11]	*Hylobates lar*	longgroup	4	1998	18	Both	S	2	1	2.000	1	6.00
[11]	*Hylobates lar*	longgroup	4	1999	19	Both	S	2	2	1.000	1	6.00
[11]	*Hylobates lar*	longgroup	4	2000	20	Both	S	2	1	2.000	1	6.00
[11]	*Hylobates lar*	longgroup	4	2001	21	Both	S	1	1	1.000	1	5.00
[11]	*Hylobates lar*	longgroup	4	2002	22	Both	S	1	1	1.000	1	4.00
[11]	*Hylobates lar*	longgroup	4	2003	23	Both	S	1	1	1.000	1	5.00
[11]	*Hylobates lar*	longgroup	4	2004	24	Both	S	1	1	1.000	1	4.00
[11]	*Hylobates lar*	longgroup	4	2005	25	Both	S	1	1	1.000	1	2.00
[11]	*Hylobates lar*	longgroup	4	2006	26	Both	S	1	1	1.000	1	3.00
[11]	*Hylobates lar*	longgroup	4	2007	27	Both	S	1	1	1.000	1	3.00
[11]	*Hylobates lar*	longgroup	4	2008	28	Both	S	1	1	1.000	1	3.00
[11]	*Hylobates lar*	longgroup	4	2009	29	Both	S	1	1	1.000	1	3.00
[11]	*Hylobates lar*	longgroup	4	2010	30	Both	S	1	1	1.000	1	4.00
[11]	*Hylobates lar*	longgroup	5	1999	1	Both	S	1	1	1.000	1	2.00
[11]	*Hylobates lar*	longgroup	5	2000	2	Both	S	1	1	1.000	1	3.00
[11]	*Hylobates lar*	longgroup	5	2001	3	Both	S	1	1	1.000	1	4.00
[11]	*Hylobates lar*	longgroup	5	2002	4	Both	S	2	1	2.000	1	3.00
[11]	*Hylobates lar*	longgroup	5	2003	5	Both	S	2	1	2.000	1	4.00
[11]	*Hylobates lar*	longgroup	5	2004	6	Both	S	2	1	2.000	1	4.00
[11]	*Hylobates lar*	longgroup	5	2005	7	Both	S	2	1	2.000	1	4.00
[11]	*Hylobates lar*	longgroup	5	2006	8	Both	S	1	1	1.000	1	4.00

Source	Taxa	Type	Group identifier	Year	Year in study sequence	Dispersal	Grouping	Ad males	Ad females	Sex ratio	N groups	Group size
[11]	*Hylobates lar*	longgroup	5	2007	9	Both	S	1	1	1.000	1	4.00
[11]	*Hylobates lar*	longgroup	5	2008	10	Both	S	1	1	1.000	1	4.00
[11]	*Hylobates lar*	longgroup	5	2009	11	Both	S	1	1	1.000	1	4.00
[11]	*Hylobates lar*	longgroup	5	2010	12	Both	S	1	1	1.000	1	5.00
[12, 13]	*Lagothrix lagotricha*	longpop	1	1987	1	Female	FF	4	6	0.670	11	12.00
[12, 13]	*Lagothrix lagotricha*	longpop	1	1988	2	Female	FF	4	6	0.670	11	15.00
[12, 13]	*Lagothrix lagotricha*	longpop	1	1989	3	Female	FF	4	6	0.670	11	17.00
[12, 13]	*Lagothrix lagotricha*	longpop	1	1990	4	Female	FF	4	7	0.590	11	20.00
[12, 13]	*Lagothrix lagotricha*	longpop	1	1991	5	Female	FF	3	8	0.380	11	20.00
[12, 13]	*Lagothrix lagotricha*	longpop	1	1992	6	Female	FF	3	8	0.380	11	21.00
[12, 13]	*Lagothrix lagotricha*	longpop	1	1993	7	Female	FF	4	8	0.500	11	20.00
[14]	*Lemur catta*	longpop	1	1987	1	Male	SFF	4.5	3.6	1.340	8	12.00
[14]	*Lemur catta*	longpop	1	1991	5	Male	SFF	4.2	5.6	0.800	9	11.30
[14]	*Lemur catta*	longpop	1	1996	10	Male	SFF	4.2	4.1	1.030	8	11.50
[14]	*Lemur catta*	longpop	1	2001	15	Male	SFF	3.2	4	0.820	9	11.00
[15]	*Macaca maura*	longpop	1	1988	1	Male	S	2	8	0.250	1	20.00
[15]	*Macaca maura*	longpop	1	1990	2	Male	S	3	10	0.330	5	22.00
[15]	*Macaca maura*	longpop	1	1991	3	Male	S	5	10	0.500	2	25.00
[15]	*Macaca maura*	longpop	1	1992	4	Male	S	4	11	0.360	7	26.00
[15]	*Macaca maura*	longpop	1	1993	5	Male	S	3	10	0.330	7	29.00
[15]	*Macaca maura*	longpop	1	1994	6	Male	S	2	10	0.200	2	26.00
[15]	*Macaca maura*	longpop	1	1995	7	Male	S	4	11	0.360	2	34.00
[15]	*Macaca maura*	longpop	1	1996	8	Male	S	5	11	0.450	2	36.00
[15]	*Macaca maura*	longpop	1	1997	9	Male	S	8	13	0.620	2	41.00
[15]	*Macaca maura*	longpop	1	1998	10	Male	S	9	12	0.750	2	44.00
[16]	*Macaca radiata*	crossgroup	1	1996	1	Male	S	1	1	1.000	1	7.00
[16]	*Macaca radiata*	crossgroup	1	1996	1	Male	S	1	2	0.500	1	6.00
[16]	*Macaca radiata*	crossgroup	1	1996	1	Male	S	1	2	0.500	1	9.00
[16]	*Macaca radiata*	crossgroup	1	1996	1	Male	S	1	3	0.330	1	7.00
[16]	*Macaca radiata*	crossgroup	1	1996	1	Male	S	1	3	0.330	1	13.00
[16]	*Macaca radiata*	crossgroup	1	1996	1	Male	S	1	3	0.330	1	12.00
[16]	*Macaca radiata*	crossgroup	1	1996	1	Male	S	1	3	0.330	1	14.00
[16]	*Macaca radiata*	crossgroup	1	1996	1	Male	S	1	3	0.330	1	12.00
[16]	*Macaca radiata*	crossgroup	1	1996	1	Male	S	1	4	0.250	1	10.00
[16]	*Macaca radiata*	crossgroup	1	1996	1	Male	S	1	4	0.250	1	13.00
[16]	*Macaca radiata*	crossgroup	1	1996	1	Male	S	1	4	0.250	1	16.00
[16]	*Macaca radiata*	crossgroup	1	1996	1	Male	S	2	1	2.000	1	8.00
[16]	*Macaca radiata*	crossgroup	1	1996	1	Male	S	2	1	2.000	1	8.00
[16]	*Macaca radiata*	crossgroup	1	1996	1	Male	S	2	4	0.500	1	16.00

Source	Taxa	Type	Group identifier	Year	Year in study sequence	Dispersal	Grouping	Ad males	Ad females	Sex ratio	N groups	Group size
[16]	*Macaca radiata*	crossgroup	1	1996	1	Male	S	3	4	0.750	1	16.00
[16]	*Macaca radiata*	crossgroup	1	1996	1	Male	S	3	5	0.600	1	18.00
[16]	*Macaca radiata*	crossgroup	1	1996	1	Male	S	4	5	0.800	1	17.00
[16]	*Macaca radiata*	crossgroup	1	1996	1	Male	S	5	6	0.830	1	30.00
[16]	*Macaca radiata*	crossgroup	1	1996	1	Male	S	5	7	0.710	1	26.00
[16]	*Macaca radiata*	crossgroup	1	1996	1	Male	S	6	5	1.200	1	37.00
[16]	*Macaca radiata*	crossgroup	1	1996	1	Male	S	6	7	0.860	1	26.00
[16]	*Macaca radiata*	longgroup	2	2000	1	Male	S	8	10	0.800	1	44.00
[16]	*Macaca radiata*	longgroup	2	2003	3	Male	S	11	11	1.000	1	52.00
[16]	*Macaca radiata*	longgroup	3	2000	1	Male	S	5	9	0.560	1	30.00
[16]	*Macaca radiata*	longgroup	3	2003	3	Male	S	7	11	0.640	1	35.00
[16]	*Macaca radiata*	longgroup	4	2000	1	Male	S	3	6	0.500	1	18.00
[16]	*Macaca radiata*	longgroup	4	2003	3	Male	S	4	6	0.670	1	32.00
[16]	*Macaca radiata*	longgroup	5	2000	1	Male	S	1	3	0.330	1	9.00
[16]	*Macaca radiata*	longgroup	5	2003	3	Male	S	1	3	0.330	1	11.00
[17]	*Macaca sylvanus*	longgroup	1	1983	1	Male	S	7	7	1.000	1	38.00
[17]	*Macaca sylvanus*	longgroup	1	1984	2	Male	S	9	9	1.000	1	47.00
[17]	*Macaca sylvanus*	longgroup	1	1985	3	Male	S	10	14	0.710	1	59.00
[17]	*Macaca sylvanus*	longgroup	1	1986	4	Male	S	16	17	0.940	1	69.00
[17]	*Macaca sylvanus*	longgroup	1	1987	5	Male	S	20	19	1.050	1	73.00
[17]	*Macaca sylvanus*	longgroup	1	1988	6	Male	S	26	23	1.130	1	88.00
[17]	*Macaca sylvanus*	longgroup	2	1989	1	Male	S	14	12	1.170	1	50.00
[17]	*Macaca sylvanus*	longgroup	2	1990	2	Male	S	15	14	1.070	1	55.00
[17]	*Macaca sylvanus*	longgroup	3	1989	1	Male	S	7	7	1.000	1	24.00
[17]	*Macaca sylvanus*	longgroup	3	1990	2	Male	S	9	7	1.290	1	30.00
[17]	*Macaca sylvanus*	longgroup	4	1989	1	Male	S	1	3	0.330	1	13.00
[17]	*Macaca sylvanus*	longgroup	4	1990	2	Male	S	6	5	1.200	1	21.00
[17]	*Macaca sylvanus*	longgroup	5	1983	1	Male	S	8	6	1.330	1	33.00
[17]	*Macaca sylvanus*	longgroup	5	1984	2	Male	S	10	8	1.250	1	41.00
[17]	*Macaca sylvanus*	longgroup	5	1985	3	Male	S	7	10	0.700	1	36.00
[17]	*Macaca sylvanus*	longgroup	5	1986	4	Male	S	7	11	0.640	1	41.00
[17]	*Macaca sylvanus*	longgroup	5	1987	5	Male	S	9	12	0.750	1	47.00
[17]	*Macaca sylvanus*	longgroup	5	1988	6	Male	S	12	9	1.330	1	46.00
[17]	*Macaca sylvanus*	longgroup	5	1989	7	Male	S	11	11	1.000	1	45.00
[17]	*Macaca sylvanus*	longgroup	5	1990	8	Male	S	13	15	0.870	1	53.00
[18]	*Pan paniscus*	longgroup	1	1977	1	Female	FF	14	15	1.000	1	58.00
[18]	*Pan paniscus*	longgroup	1	1979	3	Female	FF	16	15	0.940	1	61.00
[18]	*Pan paniscus*	longgroup	1	1980	4	Female	FF	16	16	1.000	1	68.00
[18]	*Pan paniscus*	longgroup	1	1981	5	Female	FF	20	20	1.000	1	75.00

Source	Taxa	Type	Group identifier	Year	Year in study sequence	Dispersal	Grouping	Ad males	Ad females	Sex ratio	N groups	Group size
[18]	*Pan paniscus*	longgroup	1	1983	7	Female	FF	21	18	0.860	1	72.00
[19]	*Pan paniscus*	longgroup	1	1984	8	Female	FF	6	7	0.860	1	25.00
[20]	*Pan troglodytes schweinfurthii*	longgroup	1	1987	1	Female	FF	8	12	0.670	1	40.00
[20]	*Pan troglodytes schweinfurthii*	longgroup	1	1991	4	Female	FF	12	16	0.750	1	55.00
[20]	*Pan troglodytes schweinfurthii*	longgroup	1	1996	9	Female	FF	10	16	0.630	1	50.00
[20]	*Pan troglodytes schweinfurthii*	longgroup	1	2006	19	Female	FF	11	15	0.730	1	47.00
[21]	*Pan troglodytes schweinfurthii*	longgroup	1	1998	1	Female	FF	23	45	0.510	1	142.00
[21]	*Pan troglodytes schweinfurthii*	longgroup	1	2003	6	Female	FF	25	42	0.600	1	145.00
[21]	*Pan troglodytes schweinfurthii*	longgroup	1	2009	12	Female	FF	32	44	0.730	1	165.00
[22]	*Pan troglodytes verus*	longgroup	1	1992	1	Female	FF	6	16	0.380	1	46.50
[22]	*Pan troglodytes verus*	longgroup	1	1993	2	Female	FF	5	15.5	0.320	1	41.50
[22]	*Pan troglodytes verus*	longgroup	1	1994	3	Female	FF	3	13.5	0.220	1	36.00
[22]	*Pan troglodytes verus*	longgroup	1	1995	4	Female	FF	2	11	0.180	1	32.00
[22]	*Pan troglodytes verus*	longgroup	1	1996	5	Female	FF	2	10.5	0.190	1	33.00
[22]	*Pan troglodytes verus*	longgroup	1	1997	6	Female	FF	1.5	10.5	0.140	1	32.00
[22]	*Pan troglodytes verus*	longgroup	1	1998	7	Female	FF	1.5	11	0.140	1	31.00
[22]	*Pan troglodytes verus*	longgroup	1	1999	8	Female	FF	1.5	9	0.170	1	27.00
[22]	*Pan troglodytes verus*	longgroup	1	2000	8	Female	FF	1	7	0.140	1	23.00
[22]	*Pan troglodytes verus*	longgroup	1	2001	10	Female	FF	1	6.5	0.150	1	22.00
[23]	*Papio anubis*	longgroup	1	1978	1	Male	S	14	34	0.410	1	115.00
[24]	*Papio anubis*	longgroup	1	1984	6	Male	S	14	37	0.380	1	105.00
[25]	*Papio cynocephalus*	longgroup	1	1971	1	Male	S	11	13	0.850	1	38.00
[25]	*Papio cynocephalus*	longgroup	1	1972	2	Male	S	6	12	0.500	1	32.00
[26]	*Papio cynocephalus*	longgroup	1	1979	9	Male	S	6	12	0.500	1	31.00
[24]	*Papio cynocephalus*	longgroup	1	1981	10	Male	S	6	14	0.430	1	33.00
[24]	*Papio cynocephalus*	longgroup	1	1982	11	Male	S	7	16	0.440	1	41.00
[27]	*Papio cynocephalus*	longgroup	1	1987	16	Male	S	8	18	0.440	1	55.00
[25]	*Papio cynocephalus*	longgroup	1	1971	1	Male	S	8	13	0.620	1	36.00
[25]	*Papio cynocephalus*	longgroup	1	1972	2	Male	S	8	10	0.800	1	35.00
[28]	*Papio cynocephalus*	longgroup	1	1975	5	Male	S	10	17	0.590	1	42.00
[28]	*Papio cynocephalus*	longgroup	1	1976	6	Male	S	5	14	0.360	1	37.00
[26]	*Papio cynocephalus*	longgroup	1	1979	10	Male	S	NA	NA	NA	1	44.00
[24]	*Papio cynocephalus*	longgroup	1	1981	11	Male	S	7	18	0.390	1	50.00
[24]	*Papio cynocephalus*	longgroup	1	1982	12	Male	S	9	19	0.470	1	55.00
[27]	*Papio cynocephalus*	longgroup	1	1987	17	Male	S	8	18	0.440	1	78.00
[29]	*Papio ursinus*	crossgroup	1	1998	1	Male	SFF	3	6	0.500	1	18.00
[29]	*Papio ursinus*	crossgroup	1	1998	1	Male	SFF	7	12	0.580	1	36.00
[29]	*Papio ursinus*	crossgroup	1	1998	1	Male	SFF	1	7	0.140	1	19.00
[29]	*Papio ursinus*	crossgroup	1	1998	1	Male	SFF	2	12	0.170	1	44.00

Source	Taxa	Type	Group identifier	Year	Year in study sequence	Dispersal	Grouping	Ad males	Ad females	Sex ratio	N groups	Group size
[30]	*Papio ursinus*	crossgroup	1	1999	1	Male	SFF	1	8	0.130	1	22.00
[30]	*Papio ursinus*	crossgroup	1	1999	1	Male	SFF	2	11	0.090	1	24.00
[30]	*Papio ursinus*	crossgroup	1	1999	1	Male	SFF	4	13	0.310	1	36.00
[30]	*Papio ursinus*	crossgroup	1	1999	1	Male	SFF	6	15	0.400	1	55.00
[31]	*Piliocolobus gordonorum*	longpop	1	1977	1	Both	SFF	3	9	0.330	1	23.00
[31]	*Piliocolobus gordonorum*	longpop	1	1977	1	Both	SFF	4	11	0.360	1	33.00
[31]	*Piliocolobus gordonorum*	longpop	1	1992	15	Both	SFF	1	13	0.080	1	34.00
[31]	*Piliocolobus gordonorum*	longpop	1	1992	15	Both	SFF	2	14	0.140	1	31.00
[31]	*Piliocolobus gordonorum*	longpop	1	1992	15	Both	SFF	3	11	0.270	1	26.00
[31]	*Piliocolobus gordonorum*	longpop	1	1992	15	Both	SFF	2	26	0.080	1	42.00
[31]	*Piliocolobus gordonorum*	longpop	2	1998	1	Both	SFF	1	4	0.250	1	7.00
[31]	*Piliocolobus gordonorum*	longpop	2	1998	1	Both	SFF	4	29	0.140	1	55.00
[31]	*Piliocolobus gordonorum*	longpop	2	2001	2	Both	SFF	6	15	0.400	1	40.00
[31]	*Piliocolobus gordonorum*	longpop	2	2001	2	Both	SFF	3	9	0.330	1	23.00
[31]	*Piliocolobus gordonorum*	longpop	2	2001	2	Both	SFF	5	28	0.180	1	62.00
[31]	*Piliocolobus gordonorum*	longpop	2	2000	1	Both	SFF	4	11	0.360	1	28.00
[31]	*Piliocolobus gordonorum*	longpop	2	2000	1	Both	SFF	5	17	0.290	1	38.00
[31]	*Piliocolobus gordonorum*	longpop	2	2000	1	Both	SFF	3	20	0.150	1	47.00
[31]	*Piliocolobus gordonorum*	longpop	2	2000	1	Both	SFF	5	15	0.330	1	40.00
[31]	*Piliocolobus gordonorum*	longpop	2	2000	1	Both	SFF	4	14	0.290	1	26.00
[31]	*Piliocolobus gordonorum*	longpop	2	2000	1	Both	SFF	6	16	0.380	1	40.00
[31]	*Piliocolobus gordonorum*	longpop	2	2000	1	Both	SFF	3	11	0.270	1	28.00
[31]	*Piliocolobus gordonorum*	longpop	3	1998	1	Both	SFF	2	3	0.670	1	9.00
[31]	*Piliocolobus gordonorum*	longpop	3	1998	1	Both	SFF	8	13	0.620	1	28.00
[31]	*Piliocolobus gordonorum*	longpop	3	1998	1	Both	SFF	3	11	0.270	1	24.00
[31]	*Piliocolobus gordonorum*	longpop	3	1998	1	Both	SFF	2	10	0.200	1	19.00
[31]	*Piliocolobus gordonorum*	longpop	3	1999	2	Both	SFF	1	4	0.250	1	8.00
[32]	*Piliocolobus kirkii*	longgroup	1	2003	1	Both	SFF	3	5	0.600	1	14.00
[32]	*Piliocolobus kirkii*	longgroup	1	2004	2	Both	SFF	3	6	0.500	1	15.00
[32]	*Piliocolobus kirkii*	longgroup	1	2005	3	Both	SFF	3	13	0.230	1	24.00
[32]	*Piliocolobus kirkii*	longgroup	2	2003	1	Both	SFF	3	5	0.600	1	14.00
[32]	*Piliocolobus kirkii*	longgroup	2	2004	2	Both	SFF	2	5	0.400	1	14.00
[32]	*Piliocolobus kirkii*	longgroup	3	2003	1	Both	SFF	3	6	0.500	1	16.00
[32]	*Piliocolobus kirkii*	longgroup	3	2004	2	Both	SFF	4	7	0.570	1	17.00
[32]	*Piliocolobus kirkii*	longgroup	3	2005	3	Both	SFF	4	8	0.500	1	19.00
[32]	*Piliocolobus kirkii*	longgroup	4	2003	1	Both	SFF	4	11	0.360	1	31.00
[32]	*Piliocolobus kirkii*	longgroup	4	2004	2	Both	SFF	2	9	0.220	1	31.00
[32]	*Piliocolobus kirkii*	longgroup	4	2005	3	Both	SFF	3	16	0.190	1	35.00
[32]	*Piliocolobus kirkii*	longgroup	5	2003	1	Both	SFF	3	7	0.430	1	16.00

Source	Taxa	Type	Group identifier	Year	Year in study sequence	Dispersal	Grouping	Ad males	Ad females	Sex ratio	N groups	Group size
[32]	*Piliocolobus kirkii*	longgroup	5	2004	2	Both	SFF	3	7	0.430	1	18.00
[32]	*Piliocolobus kirkii*	longgroup	5	2005	3	Both	SFF	2	7	0.290	1	22.00
[32]	*Piliocolobus kirkii*	longgroup	6	2003	1	Both	SFF	3	10	0.330	1	22.00
[32]	*Piliocolobus kirkii*	longgroup	6	2004	2	Both	SFF	2	6	0.330	1	18.00
[32]	*Piliocolobus kirkii*	longgroup	6	2005	3	Both	SFF	2	9	0.220	1	23.00
[32]	*Piliocolobus kirkii*	longgroup	7	2003	1	Both	SFF	2	5	0.400	1	12.00
[32]	*Piliocolobus kirkii*	longgroup	7	2004	2	Both	SFF	2	6	0.330	1	13.00
[32]	*Piliocolobus kirkii*	longgroup	8	2003	1	Both	SFF	1	4	0.250	1	7.00
[32]	*Piliocolobus kirkii*	longgroup	8	2004	2	Both	SFF	1	2	0.500	1	5.00
[32]	*Piliocolobus kirkii*	longgroup	8	2005	3	Both	SFF	1	6	0.170	1	9.00
[32]	*Piliocolobus kirkii*	longgroup	9	2004	1	Both	SFF	2	2	1.000	1	7.00
[32]	*Piliocolobus kirkii*	longgroup	9	2005	2	Both	SFF	1	4	0.250	1	7.00
[32]	*Piliocolobus kirkii*	longgroup	10	2003	1	Both	SFF	3	15	0.200	1	22.00
[32]	*Piliocolobus kirkii*	longgroup	10	2004	2	Both	SFF	2	8	0.250	1	21.00
[32]	*Piliocolobus kirkii*	longgroup	10	2005	3	Both	SFF	3	18	0.170	1	35.00
[32]	*Piliocolobus kirkii*	longgroup	11	2003	1	Both	SFF	2	7	0.290	1	16.00
[32]	*Piliocolobus kirkii*	longgroup	11	2004	2	Both	SFF	2	8	0.250	1	21.00
[32]	*Piliocolobus kirkii*	longgroup	11	2005	3	Both	SFF	2	9	0.220	1	24.00
[33]	*Piliocolobus tephrosceles*	crossgroup	1	1998	1	Female	SFF	7	20	0.350	1	48.00
[33]	*Piliocolobus tephrosceles*	crossgroup	1	1998	1	Female	SFF	6	8	0.750	1	24.00
[34]	*Piliocolobus tephrosceles*	longgroup	1	1970	1	Female	SFF	3	7	0.430	1	22.00
[34]	*Piliocolobus tephrosceles*	longgroup	1	1971	2	Female	SFF	3	7	0.430	1	19.00
[34]	*Piliocolobus tephrosceles*	longgroup	1	1972	3	Female	SFF	4	7	0.570	1	19.00
[34]	*Piliocolobus tephrosceles*	longgroup	1	1973	4	Female	SFF	4	8	0.500	1	20.00
[34]	*Piliocolobus tephrosceles*	longgroup	1	1974	5	Female	SFF	4	8	0.500	1	20.00
[34]	*Piliocolobus tephrosceles*	longgroup	1	1975	6	Female	SFF	4	9	0.440	1	25.00
[34]	*Piliocolobus tephrosceles*	longgroup	1	1976	7	Female	SFF	5	10	0.500	1	NA
[34]	*Piliocolobus tephrosceles*	longgroup	1	1977	8	Female	SFF	6	10	0.600	1	NA
[34]	*Piliocolobus tephrosceles*	longgroup	1	1978	9	Female	SFF	4	12	0.330	1	37.00
[34]	*Piliocolobus tephrosceles*	longgroup	1	1979	10	Female	SFF	4	14	0.290	1	NA
[34]	*Piliocolobus tephrosceles*	longgroup	1	1980	11	Female	SFF	3	15	0.200	1	NA
[34]	*Piliocolobus tephrosceles*	longgroup	1	1981	12	Female	SFF	3	15	0.200	1	NA
[34]	*Piliocolobus tephrosceles*	longgroup	1	1982	13	Female	SFF	4	14	0.290	1	NA
[34]	*Piliocolobus tephrosceles*	longgroup	1	1983	14	Female	SFF	1	10	0.100	1	NA
[34]	*Piliocolobus tephrosceles*	longgroup	1	1984	15	Female	SFF	1	4	0.250	1	8.00
[34]	*Piliocolobus tephrosceles*	longgroup	1	1985	16	Female	SFF	2	2	1.000	1	8.00
[34]	*Piliocolobus tephrosceles*	longgroup	2	1978	1	Female	SFF	9	20	0.450	1	50.00
[34]	*Piliocolobus tephrosceles*	longgroup	2	1979	2	Female	SFF	7	18	0.390	1	NA
[34]	*Piliocolobus tephrosceles*	longgroup	2	1980	3	Female	SFF	7	17	0.410	1	NA

Source	Taxa	Type	Group identifier	Year	Year in study sequence	Dispersal	Grouping	Ad males	Ad females	Sex ratio	N groups	Group size
[34]	*Piliocolobus tephrosceles*	longgroup	2	1981	4	Female	SFF	5	10	0.500	1	NA
[34]	*Piliocolobus tephrosceles*	longgroup	2	1982	5	Female	SFF	6	10	0.600	1	NA
[34]	*Piliocolobus tephrosceles*	longgroup	2	1983	6	Female	SFF	3	11	0.270	1	25.00
[34]	*Piliocolobus tephrosceles*	longgroup	3	1976	1	Female	SFF	4	2	2.000	1	7.00
[34]	*Piliocolobus tephrosceles*	longgroup	3	1977	2	Female	SFF	3	2	1.500	1	NA
[34]	*Piliocolobus tephrosceles*	longgroup	3	1978	3	Female	SFF	3	2	1.500	1	NA
[34]	*Piliocolobus tephrosceles*	longgroup	3	1979	4	Female	SFF	3	1	3.000	1	17.00
[34]	*Piliocolobus tephrosceles*	longgroup	3	1980	5	Female	SFF	5	1	0.200	1	17.00
[35, 36]	*Semnopithecus entellus*	longgroup	1	1992	1	Male	S	2	4	0.500	1	10.00
[35, 36]	*Semnopithecus entellus*	longgroup	1	1993	2	Male	S	2	4	0.500	1	12.00
[35, 36]	*Semnopithecus entellus*	longgroup	1	1994	3	Male	S	2	2	1.000	1	9.00
[35, 36]	*Semnopithecus entellus*	longgroup	1	1995	4	Male	S	1	3	0.333	1	12.00
[35, 36]	*Semnopithecus entellus*	longgroup	1	1996	5	Male	S	1	4	0.250	1	13.00
[35, 36]	*Semnopithecus entellus*	longgroup	2	1992	1	Male	S	3	7	0.429	1	17.00
[35, 36]	*Semnopithecus entellus*	longgroup	2	1993	2	Male	S	4	7	0.571	1	21.00
[35, 36]	*Semnopithecus entellus*	longgroup	2	1994	3	Male	S	2	7	0.286	1	18.00
[35, 36]	*Semnopithecus entellus*	longgroup	2	1995	4	Male	S	1	9	0.111	1	15.00
[35, 36]	*Semnopithecus entellus*	longgroup	2	1996	5	Male	S	1	8	0.125	1	15.00
[35, 36]	*Semnopithecus entellus*	longgroup	3	1992	1	Male	S	1	8	0.125	1	18.00
[35, 36]	*Semnopithecus entellus*	longgroup	3	1993	2	Male	S	1	5	0.200	1	16.00
[35, 36]	*Semnopithecus entellus*	longgroup	3	1994	3	Male	S	1	6	0.167	1	19.00
[35, 36]	*Semnopithecus entellus*	longgroup	3	1995	4	Male	S	3	6	0.500	1	16.00
[35, 36]	*Semnopithecus entellus*	longgroup	3	1996	5	Male	S	2	6	0.333	1	10.00
[35, 36]	*Semnopithecus entellus*	longgroup	4	1992	1	Male	S	2	4	0.500	1	17.00
[35, 36]	*Semnopithecus entellus*	longgroup	4	1993	2	Male	S	1	2	0.500	1	11.00
[35, 36]	*Semnopithecus entellus*	longgroup	4	1994	3	Male	S	1	3	0.333	1	11.00
[35, 36]	*Semnopithecus entellus*	longgroup	4	1995	4	Male	S	1	3	0.333	1	13.00
[35, 36]	*Semnopithecus entellus*	longgroup	4	1996	5	Male	S	4	8	0.500	1	19.00
[35, 36]	*Semnopithecus entellus*	longgroup	5	1992	1	Male	S	2	9	0.222	1	23.00
[35, 36]	*Semnopithecus entellus*	longgroup	5	1993	2	Male	S	2	9	0.222	1	28.00
[35, 36]	*Semnopithecus entellus*	longgroup	5	1994	3	Male	S	4	7	0.571	1	24.00
[35, 36]	*Semnopithecus entellus*	longgroup	5	1995	4	Male	S	2	9	0.222	1	24.00
[35, 36]	*Semnopithecus entellus*	longgroup	5	1996	5	Male	S	3	6	0.500	1	20.00
[35, 36]	*Semnopithecus entellus*	longgroup	6	1992	1	Male	S	5	13	0.385	1	34.00
[35, 36]	*Semnopithecus entellus*	longgroup	6	1993	2	Male	S	5	12	0.417	1	31.00
[35, 36]	*Semnopithecus entellus*	longgroup	6	1994	3	Male	S	3	14	0.214	1	31.00
[35, 36]	*Semnopithecus entellus*	longgroup	6	1995	4	Male	S	5	14	0.357	1	30.00
[35, 36]	*Semnopithecus entellus*	longgroup	6	1996	5	Male	S	4	12	0.333	1	26.00
[35, 36]	*Semnopithecus entellus*	longgroup	6	1997	6	Male	S	3	15	0.200	1	31.00

Source	Taxa	Type	Group identifier	Year	Year in study sequence	Dispersal	Grouping	Ad males	Ad females	Sex ratio	N groups	Group size
[35, 36]	*Semnopithecus entellus*	longgroup	7	1991	1	Male	S	1	9	0.111	1	20.00
[35, 36]	*Semnopithecus entellus*	longgroup	7	1992	2	Male	S	3	9	0.333	1	21.00
[35, 36]	*Semnopithecus entellus*	longgroup	7	1993	3	Male	S	3	9	0.333	1	25.00
[35, 36]	*Semnopithecus entellus*	longgroup	7	1994	4	Male	S	3	5	0.600	1	18.00
[35, 36]	*Semnopithecus entellus*	longgroup	7	1995	5	Male	S	3	4	0.750	1	19.00
[35, 36]	*Semnopithecus entellus*	longgroup	7	1996	6	Male	S	2	5	0.400	1	18.00
[35, 36]	*Semnopithecus entellus*	longgroup	8	1992	1	Male	S	4	12	0.333	1	28.00
[35, 36]	*Semnopithecus entellus*	longgroup	8	1993	2	Male	S	4	12	0.333	1	31.00
[35, 36]	*Semnopithecus entellus*	longgroup	8	1994	3	Male	S	4	8	0.500	1	25.00
[35, 36]	*Semnopithecus entellus*	longgroup	8	1995	4	Male	S	4	7	0.571	1	23.00
[35, 36]	*Semnopithecus entellus*	longgroup	8	1996	5	Male	S	2	8	0.250	1	22.00
[35, 36]	*Semnopithecus entellus*	longgroup	9	1992	1	Male	S	1	2	0.500	1	10.00
[35, 36]	*Semnopithecus entellus*	longgroup	9	1993	2	Male	S	1	2	0.500	1	8.00
[35, 36]	*Semnopithecus entellus*	longgroup	9	1994	3	Male	S	2	2	1.000	1	9.00
[35, 36]	*Semnopithecus entellus*	longgroup	9	1995	4	Male	S	4	2	2.000	1	10.00
[35, 36]	*Semnopithecus entellus*	longgroup	9	1996	5	Male	S	3	2	1.500	1	9.00
[35, 36]	*Semnopithecus entellus*	longgroup	10	1992	1	Male	S	2	6	0.333	1	18.00
[35, 36]	*Semnopithecus entellus*	longgroup	10	1993	2	Male	S	1	5	0.200	1	13.00
[35, 36]	*Semnopithecus entellus*	longgroup	10	1994	3	Male	S	1	6	0.167	1	14.00
[35, 36]	*Semnopithecus entellus*	longgroup	10	1995	4	Male	S	1	6	0.167	1	14.00
[35, 36]	*Semnopithecus entellus*	longgroup	10	1996	5	Male	S	1	6	0.167	1	17.00

Legend: Type: longgroup=longitudinal group, longpop=longitudinal population, crosspop=cross population; **Group identifier:** for longgroup studies, an integer identifying separate groups, set to one for other studies; **Year in study sequence:** the year of the study, starting with year 1 for each group or population; **Grouping:** S=stable, FF=fission-fusion, SFF=sometimes fission-fusion; **Ad males and Ad females:** numbers of adult males and females per group; **Sex ratio:** Ad males divided by Ad females; **Group size:** for longpop studies, this is the average group size in a given year; See text for definitions.

Sources: See Table S3.
