# Supplementary material for: Behavioral Flexibility and the Evolution of Primate Social States
Source: PLoS One. 2014 Dec 3;9(12):e114099. doi: 10.1371/journal.pone.0114099 (PMC4254976; doi:10.1371/journal.pone.0114099)
Supplement: Table S3 — References to Table S1 and Table S2. (PDF) [file pone.0114099.s003.pdf]

Table S3: References to Table S1 and Table S2.

Source	Reference
[1]	Fedigan, L.M. & Jack, K.M. 2012 Tracking neotropical monkeys in Santa Rosa: Lessons from a regenerating Costa Rican dry forest. In *Long-term field studies of primates* (eds. P.M. Kappeler & D. Watts), pp. 165-184. Berlin, Springer.
[2]	Rudran, R. & Fernandez-Duque, E. 2003 Demographic changes over thirty years in a red howler population in Venezuela. *International Journal of Primatology* **24**, 925-948.
[3]	Strier, K.B. & Mendes, S.L. 2012 The northern muriqui (*Brachyteles hypoxanthus*): Lessons on behavioral plasticity and population dynamics from a critically endangered species. In *Long-term field studies of primates* (eds. P.M. Kappeler & D. Watts), pp. 125-140. Berlin, Springer.
[4]	Struhsaker, T.T. 1967 Ecology of vervet monkeys (*Cercopithecus aethiops*) in the Masai-Amboseli game reserve, Kenya. *Ecology* **48**, 892-904.
[5]	Struhsaker, T.T. 1973 A recensus of vervet monkeys in the Masai-Amboseli Game Reserve, Kenya. *Ecology* **54**, 930-932.
[6]	Lee, P.C. 1984 Ecological constraints on the social development of vervet monkeys. *Behaviour* **91**, 245-262.
[7]	Lee, P.C. & Hauser, M.D. 1988 Long-term consequences of changes in territory quality on feeding and reproductive strategies of vervet monkeys. *Journal of Animal Ecology* **67**, 347-358.
[8]	Hauser, M.D., Cheney, D.L. & Seyfarth, R.M. 1986 Group extinction and fusion in free-ranging vervet monkeys. *American Journal of Primatology* **11**, 63-77.
[9]	Isbell, L.A., Cheney, D.L. & Seyfarth, R.M. 1991 Group fusions and minimum group sizes in vervet monkeys (*Cercopithecus aethiops*) *American Journal of Primatology* **25**, 57-65.
[10]	Gray, M., Roy, J., Vigilant, L., Fawcett, K., Basabose, A., Cranfield, M., Uwingeli, P., Mburunumwe, I., Kagoda, E. & Robbins, M.M. 2013 Genetic census reveals increased but uneven growth of a critically endangered mountain gorilla population. . *Biological Conservation* **158**, 230-238.
[11]	Reichard, U.H., Ganpanakngan, M. & Barelli, C. 2012 White-handed gibbons of Khao Yai: Social flexibility, complex reproductive strategies, and a slow life history. In *Long-term field studies of primates* (eds. P.M. Kappeler & D. Watts),

pp. 237-258. Berlin, Springer.

[12] Stevenson, P.R., Quinones, M.J. & Ahumada, J.A. 1994 Ecological strategies of woolly monkeys (*Lagothrix lagotricha*) at Tinigua National Park, Colombia. *American Journal of Primatology* **32**, 123-140.

[13] Stevenson, P.R. 2006 Activity and ranging patterns of Colombian woolly monkeys in north-western Amazonia. *Primates* **47**, 239-247.

[14] Koyama, N., Nakamichi, M., Ichino, S. & Takahata, Y. 2002 Population and social dynamics changes in ring-tailed lemur troops at Berenty, Madagascar between 1989–1999. *Primates* **43**, 291–314.

[15] Okamoto, K., Matsumura, S. & Watanabe, K. 2000 Life history and demography of wild moor macaques (*Macaca maurus*): summary of ten years of observations. *American Journal of Primatology* **52**, 1-11.

[16] Sinha, A. 2005 Not in their genes: phenotypic flexibility, behavioural traditions and cultural evolution in wild bonnet macaques. *Journal of Biosciences* **30**, 51-64.

[17] Menard, N. & Vallett, D. 1996 Demography and ecology of Barbary macaques (*Macaca sylvanus*) in two different habitats. . In *Evolution and ecology of macaque societies* (eds. J.E. Fa & D.G. Lindberg), pp. 06-131. Cambridge, Cambridge University Press.

[18] Kano, T. 1992 *The last ape*. Stanford, CA, Stanford University Press.

[19] Furuichi, T., Idani, G.I., Ihobe, H., Hashimoto, C., Tashiro, Y., Sakamaki, T., Mulavwa, M.N., Yangozene, K. & Kuroda, S. 2012 Long-term studies on wild bonobos at Wamba, Luo Scientific Reserve, DR Congo: towards the understanding of female life history in a male-philopatric species. In *Long-term field studies of primates* (eds. P.M. Kappeler & D. Watts), pp. 413-433. Berlin, Springer.

[20] Wrangham, R.W. 2000 Why are male chimpanzees more gregarious than mothers? A scramble competition hypothesis. In *Primate males* (ed. P.M. Kappeler), pp. 248-258. Cambridge, Cambridge University Press.

[21] Lwanga, J.S., Struhsaker, T.T., Struhsaker, P.J., Butynski, T.M. & Mitani, J.C. 2011 Primate population dynamics over 32.9 years at Ngogo, Kibale National Park, Uganda. *Am J Primatol* **73**, 997-1011. (doi:10.1002/ajp.20965).

[23] Smuts, B. & Nicolson, N. 1989 Reproduction in wild female olive baboons. *American Journal of Primatology* **19**, 229-246.

[24] Noe, R. 1989 Coalition formation among male baboons. Utrecht, University of Utrecht.

[25] Hausfater, G. 1975 *Dominance and reproduction in baboons (Papio cynocephalus)*. Zurich, S. Karger.

[26] Stacey, P.B. 1986 Group size and foraging efficiency in yellow baboons. *Behavioral Ecology and Sociobiology,* **18**, 175-187.

[27] Alberts, S.C. & Altmann, J. 1995 Balancing costs and opportunities: dispersal in male baboons. *American Naturalist,* **145**, 279-306.

[28] Altmann, J. 1980 *Baboon mothers and infants*. Chicago, University of Chicago Press.

[29] Henzi, P. & Barrett, L. 2003 Evolutionary ecology, sexual conflict, and behavioral differentiation among baboon populations *Evolutionary Anthropology* **12**, 217-230.

[30] Cowlishaw, G. 1999 Ecological and social determinants of spacing behaviour in desert baboon groups. *Behavioral Ecology and Sociobiology* **45**, 67-77.

[31] Struhsaker, T.T., Marshall, A.R., Detwiler, K., Siex, K., Ehardt, C., Lisbjerg, D.D. & Butynski, T.M. 2004 Demographic variation among Udzungwa red colobus in relation to gross ecological and sociological parameters. *International Journal of Primatology* **25**, 615-658.

[32] Nowak, K. 2007 Behavioral flexibility and demography of *Procolobus kirkii* across floristic and disturbance gradients. Cambridge, University of Cambridge.

[33] Chapman, C.A. & Chapman, L.J. 2000 Constraints on group size in red colobus and red-tailed guenons: examining the generality of the ecological constraints model. *International Journal of Primatology* **21**, 565-585.

[34] Struhsaker, T.T. 2000 Variation in adult sex ratio of red colobus monkey social groups: implications for interspecific comparisons. In *Primate males* (ed. P.M. Kappeler), pp. 108-119. Cambridge, Cambridge University Press.

[35] Borries, C. 2000 Male dispersal and mating season influxes in Hanuman langurs living in multi-male groups. In *Primate males* (ed. P.M. Kappeler), pp. 146-158. Cambridge, Cambridge University Press.

[36] Borries, C. & Koenig, A. Pers comm.
